# Ruthenium-based olefin metathesis catalysts with monodentate unsymmetrical NHC ligands

**DOI:** 10.3762/bjoc.14.292

**Published:** 2018-12-28

**Authors:** Veronica Paradiso, Chiara Costabile, Fabia Grisi

**Affiliations:** 1 Dipartimento di Chimica e Biologia “Adolfo Zambelli”, Università di Salerno, Via Giovanni Paolo II 132, I-84084 Fisciano, Salerno, Italy

**Keywords:** ligand design, olefin metathesis, ruthenium catalysts, selectivity, unsymmetrical N-heterocyclic carbenes

## Abstract

An overview on the catalytic properties of ruthenium complexes for olefin metathesis bearing monodentate unsymmetrical N-heterocyclic diaminocarbene ligands is provided. The non-symmetric nature of these NHC architectures strongly influences activity and selectivity of the resulting catalysts. The main achievements that have been accomplished in significant areas of olefin metathesis up to the current state of research are discussed.

## Introduction

**Figure 1:**
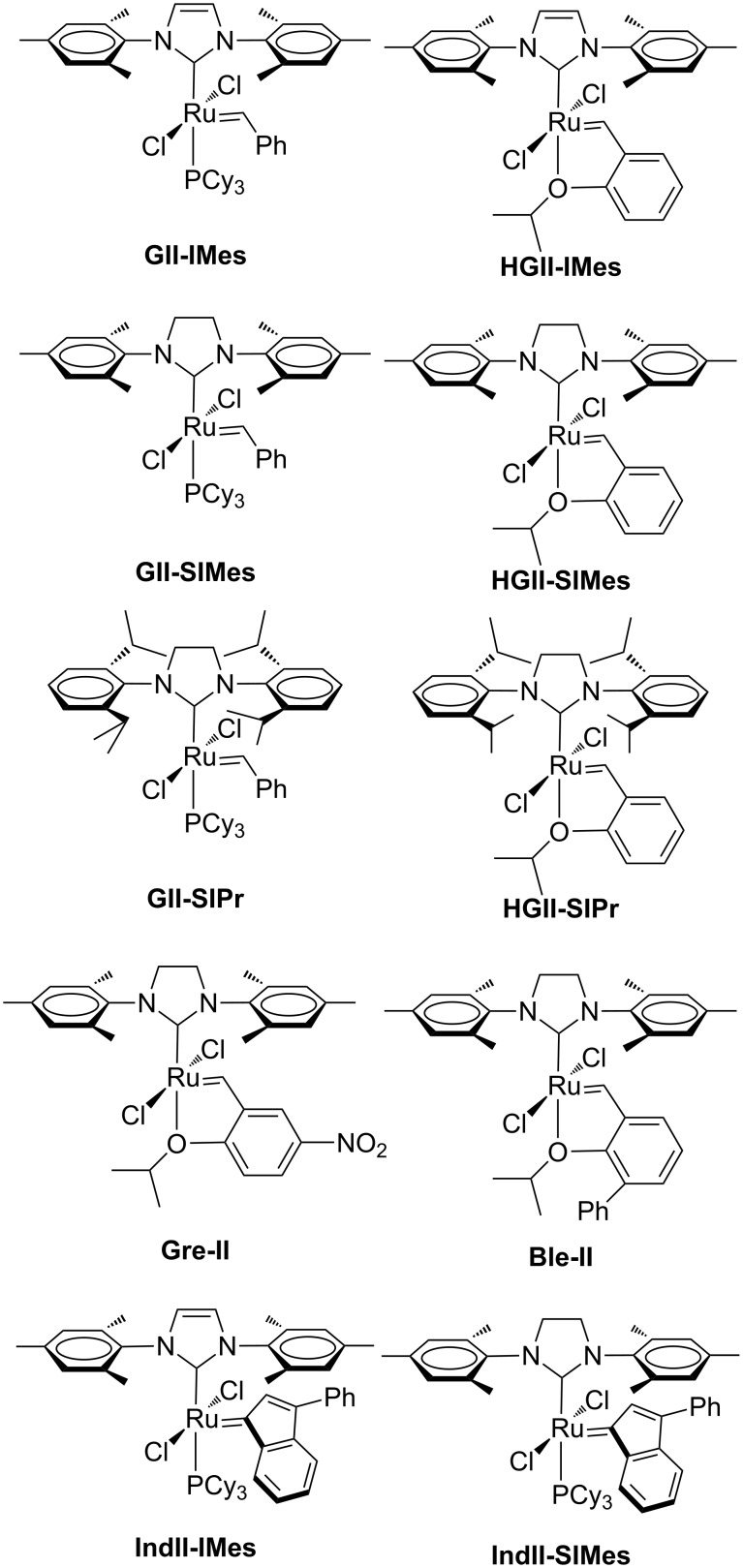
Second-generation Grubbs (**GII**), Hoveyda (**HGII**), Grela (**Gre**-**II**), Blechert (**Ble**-**II**) and indenylidene-type (**IndII**) catalysts with symmetrical NHCs.

The transition metal-catalyzed olefin metathesis reaction is an indispensable synthetic tool for the construction of new carbon–carbon double bonds in various applications in both organic and polymer chemistry [1–2]. The great popularity of this methodology is mainly related to the development of well-defined ruthenium alkylidene catalysts with high air and moisture stability and functional group tolerance. Among them, ruthenium olefin metathesis complexes bearing N-heterocyclic carbene (NHC) ligands, known as second generation catalysts (Figure 1), have shown improved catalytic efficiency over other metathesis catalysts [3–4].

Moreover, their catalytic properties can be finely modulated through variation of the steric and electronic properties of the NHC ligand. Significant advances in ruthenium metathesis catalyst design have been achieved by the introduction of unsymmetrically substituted NHC (uNHC) ligands, namely presenting different substituents at the nitrogen atoms. They offer the possibility of strongly influencing the reactivity and selectivity of the resulting catalysts by creating different steric and/or electronic environments around the metal center. Indeed, ruthenium complexes coordinated with this kind of ligands can be easily tailored for challenging or specific metathesis applications in which their symmetrical counterparts fail or show poor efficiency [5–6]. Moreover, the use of catalysts incorporating bidentate unsymmetrical NHCs has allowed for significant enhancements in the field of both asymmetric and *Z*-selective olefin metathesis reactions [7–9].

The aim of the present review is to provide a description of the catalytic behavior of ruthenium complexes bearing monodentate five-membered uNHCs. A special focus is given to the more recent advancements in the development of such unsymmetrical architectures for targeted metathesis applications.

Ruthenium complexes with NHCs presenting alternative heteroatoms, such as thiazol-2-ylidene ligands [10], or those containing one nitrogen substituent, such as the series of cyclic (alkyl) (amino) carbenes (CAACs) introduced by Bertrand et al. [11], are not included in this survey.

## Review

### Ruthenium catalysts coordinated with *N*-aryl, *N’*-aryl NHCs

**Figure 2:**
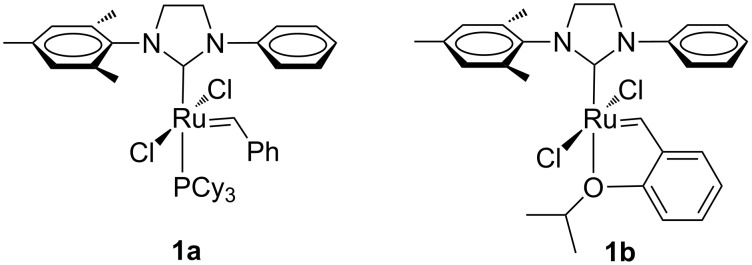
Grubbs (**1a**) and Hoveyda-type (**1b**) complexes with *N*-phenyl, *N’*-mesityl NHCs.

**Figure 3:**
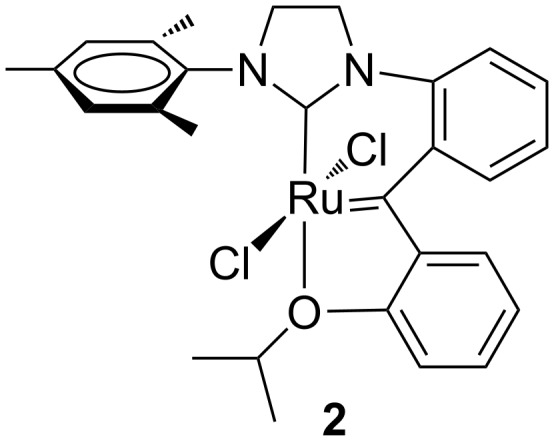
C–H insertion product **2**.

The first ruthenium complexes with monodentate NHC ligands bearing unsymmetrical *N*-aryl, *N’*-aryl groups were reported by Blechert [12], who synthesized Grubbs and Hoveyda-type complexes with *N*-phenyl, *N’*-mesityl NHC substituents (**1a**,**b** in Figure 2). Both complexes were air stable, but in CH_2_Cl_2_ solution complex **1b** converted completely within a few hours into complex **2** due to the formation of an intramolecular carbene–arene bond between the benzylidene carbon atom and the *ortho* position of the *N*-phenyl ligand (Figure 3). According to the authors, the mechanism of the reaction that occurs only in the presence of oxygen, involves a pericyclic reaction followed by an irreversible oxidation step, and, finally, a rearomatization.

**Figure 4:**
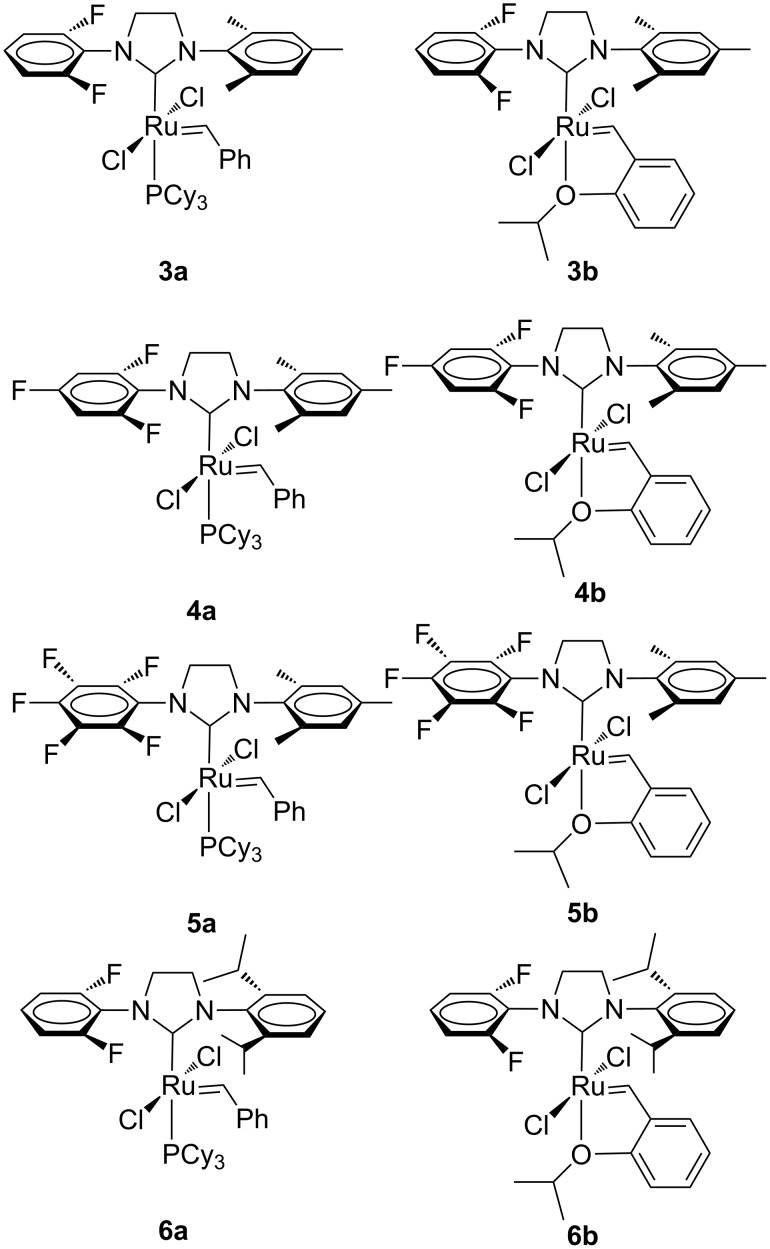
Grubbs (**3a**–**6a**) and Hoveyda-type (**3b**–**6b**) complexes with *N*-fluorophenyl, *N’*-aryl NHCs.

To avoid the C–H activation of aryl-substituted NHC ligands the corresponding *ortho* positions have to be substituted by different groups. Indeed, almost contemporaneously, Grubbs et al. reported on the synthesis of a family of corresponding *ortho*-substituted *N*-fluorophenyl, *N’*-aryl NHC Ru complexes (Figure 4) [13–14].

**Scheme 1:**
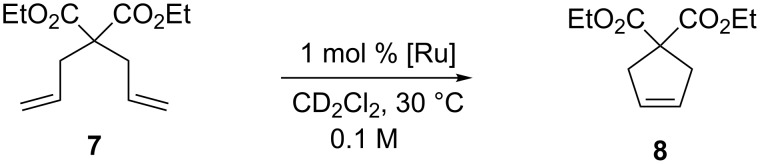
RCM of diethyl diallylmalonate (**7**).

The behavior of this catalyst family was tested in the RCM of diethyl diallylmalonate (**7**, Scheme 1) and compared with that of **GII-SIMes** and **HGII-SIMes**.

Interestingly, catalysts **3a** and **4a** clearly outperformed **GII-SIMes**, with catalyst **4a** emerging as the most efficient of all (>97% conversion in 9 min). Complex **5a** showed a higher initiation rate with respect to **GII-SIMes**, but eventually was found to be less efficient due to a decrease in its catalytic activity related to concomitant decomposition. As for Hoveyda-type catalysts **3b**, **4b** and **5b**, they all disclosed lower activity than the parent complex **HGII-SIMes**, with catalyst **5b** being the least efficient of all in this series (>97% conversion in 100 min). Finally, **6a** as well as the phosphine-free **6b** showed to be very poor olefin metathesis catalysts.

**Scheme 2:**
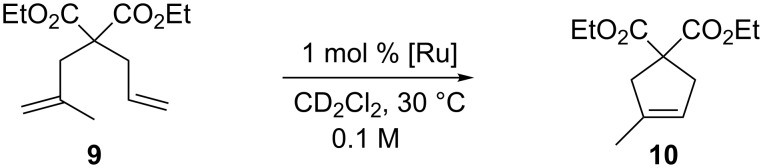
RCM of diethyl allylmethallylmalonate (**9**).

Enhanced catalytic performances, with respect to **GII-SIMes**, were previously reported also for symmetrical NHC bearing *o*-fluorinated aryl groups. Possibly the presence of a Ru–F interaction is responsible for the positive impact on the reaction rates [15]. Similar results were observed in the RCM of the more hindered diethyl allylmethallylmalonate (**9**, Scheme 2), where **3a** and **4a** behaved as the most efficient catalysts.

**Scheme 3:**
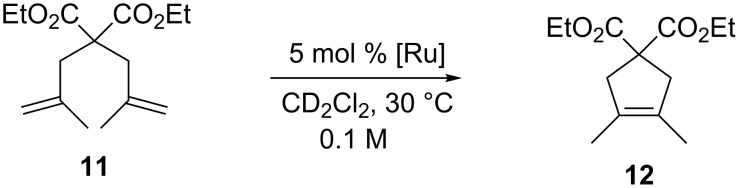
RCM of diethyl dimethallylmalonate (**11**).

Even in the challenging formation of tetrasubstituted olefin **12** via RCM (Scheme 3), catalysts **3a** and **4a** gave the best performances leading to 30% and 21% conversion, respectively, in four days.

**Scheme 4:**
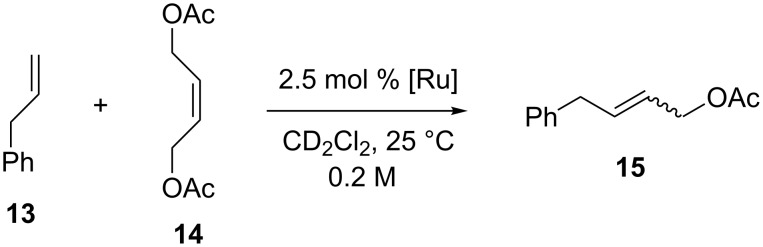
CM of allylbenzene (**13**) with *cis*-1,4-diacetoxy-2-butene (**14**).

In the CM of allylbenzene (**13**) with *cis*-1,4-diacetoxy-2-butene (**14**, Scheme 4), the fluorinated complexes **3a**–**5a** and **3b**–**5b** exhibited activities comparable to **GII-SIMes** and **HGII-SIMes**, showing higher *Z*-selectivity at conversions above 60%. For example, catalyst **GII-SIMes** affords an *E/Z* ratio of ~10 at 79% conversion, whereas catalysts **3**–**5** gave an *E/Z* ratio of about 5.5 at the same conversion.

**Scheme 5:**
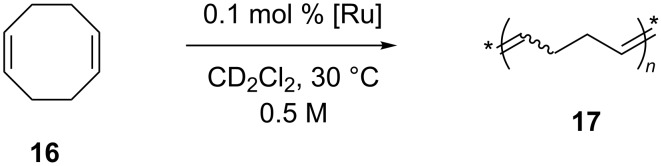
ROMP of 1,5-cyclooctadiene (**16**).

As for the ROMP of **16** (Scheme 5), **GII-SIMes** and **4a** displayed the highest activity with similar reactivity.

In the attempt to rationalize the catalytic performances of this family of *N*-fluorophenyl complexes the related [Rh(CO)_2_Cl(NHC)] complexes were synthesized. Unfortunately the shifts of the CO stretching frequencies showed that no correlation between the catalytic performances of Ru-catalysts and electronic properties of the corresponding NHC ligand is found.

**Figure 5:**
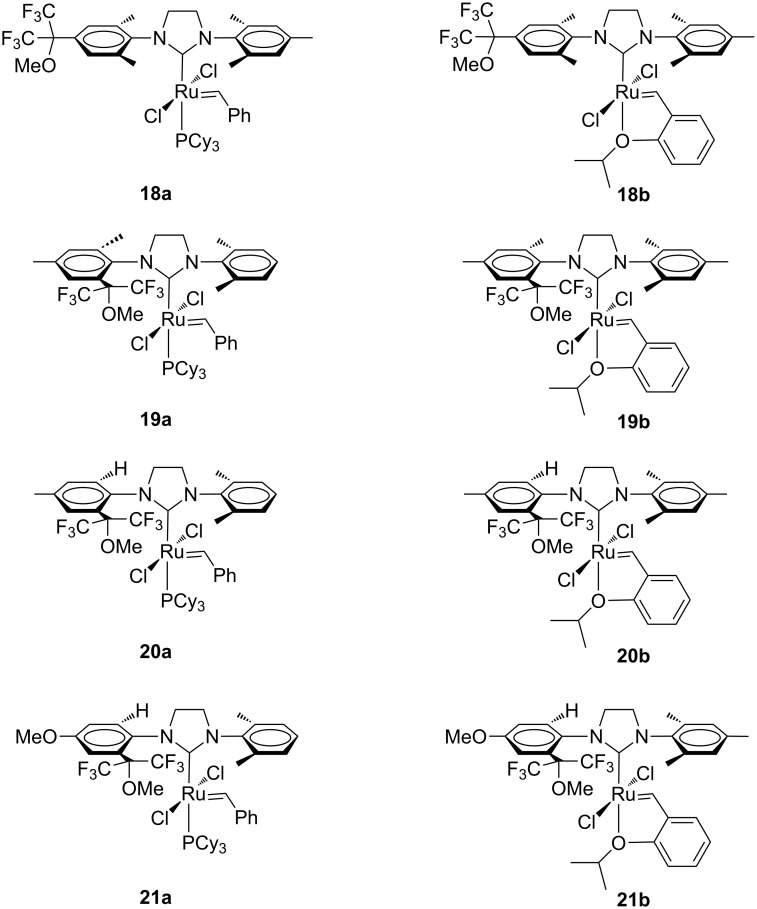
Grubbs (**18a**–**21a**) and Hoveyda-type (**18b**–**21b**) catalysts bearing uNHCs with a hexafluoroisopropylalkoxy [(CF_3_)_2_(OR)-C] group in one of the *N*-aryl substituents.

More recently, Osypov and co-workers introduced a new family of Grubbs (**18a**–**21a**) and Hoveyda-type (**18b**–**21b**) catalysts bearing unsymmetrical NHC ligands with one of the *N*-aryl substituents presenting a hexafluoroisopropylalkoxy [(CF_3_)_2_(OR)-C] group (Figure 5) [16–17].

Catalysts **18a** and **19a** showed efficiencies comparable to **GII-SIMes** and **HGII-SIMes** in the RCM of substrate **7** (Scheme 1), giving full conversion within 30 minutes, whereas the corresponding Hoveyda-type complexes **18b** and **19b** presented a more pronounced initiation period, giving good conversions in much longer reaction time (2–4 h) [16]. A similar trend was observed in the RCM of **9** (Scheme 2), but reaction rates were lower in all cases. As for **20a** and **21a**, the initiation rates in the RCM of **7** were observed to be faster than **GII-SIMes**, **HGII-SIMes** and **19a**, while the initiation rates of **20b** and **21b** were lower than **GII-SIMes** and **HGII-SIMes**, but superior to **19b**, resulting in 90% conversion within 3 hours [17]. No relevant differences in the catalyst reactivity were observed for the CM of **13** and **14** (Scheme 4).

As a novel application of *N*-aryl, *N’*-aryl unsymmetrical ruthenium complexes in enantioselective catalysis, Grela and Schmidt very recently reported on the first example of a helically chiral Hoveyda-type metathesis complex. This catalyst, bearing a mesityl and a helicene as the aryl groups, was preliminary examined in some model asymmetric metathesis transformations and showed promising levels of enantioselectivity. Further studies on the development of this new concept for enantioinduction are still ongoing [18].

### Ruthenium catalysts coordinated with *N-*alkyl, *N’*-aryl NHCs

#### *N*-Alkyl-substituents possessing no functionalities or heteroatoms

**Figure 6:**
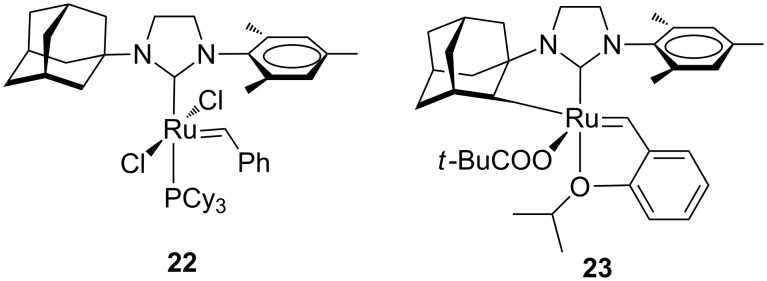
A Grubbs-type complex with an *N*-adamantyl, *N*’-mesityl NHC **22** and the Hoveyda-type complex with a chelating *N*-adamantyl, *N*’-mesityl NHC **23**.

Unsymmetrical *N*-alkyl, *N’*-aryl NHC frameworks were initially developed in order to improve the catalytic activity of ruthenium-based complexes through enhanced electron-donating ability and different steric bulk of the NHC ligand. Mol et al. introduced complex **22** (Figure 6) in which one of the mesityl groups from **GII-SIMes** was replaced by the sterically more encumbered adamantyl group [19].

**Figure 7:**
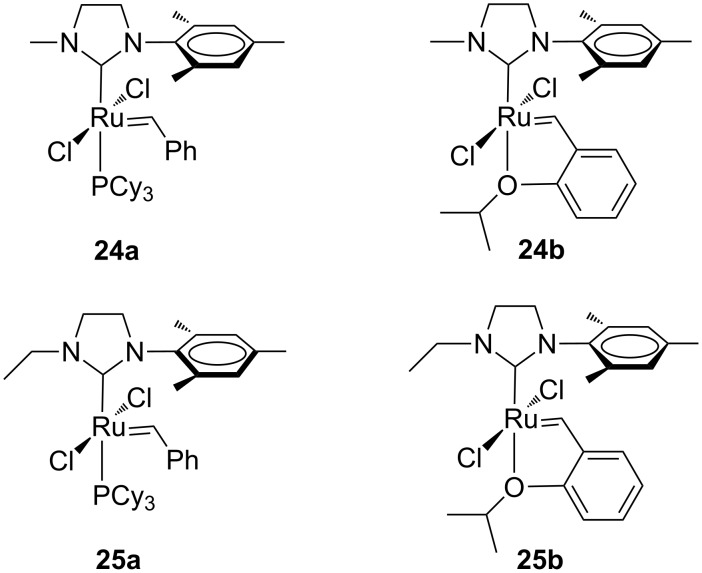
Grubbs (**24a** and **25a**) and Hoveyda-type (**24b** and **25b**) complexes with *N*-alkyl, *N’*-mesityl NHCs.

However, no beneficial effect on the catalytic activity was observed. Indeed complex **22** revealed a very poor olefin metathesis catalyst, likely as a consequence of the excessive steric hindrance of the adamantyl moiety at the ruthenium center. It is worth to underline that the first *Z*-selective ruthenium catalyst (**23**, Figure 6), developed by Grubbs and co-workers, is based on a chelating NHC ligand that is derived from an intramolecular carboxylate-driven C–H bond insertion of the adamantyl N-substituent of the same NHC ligand in complex **22** [20]. Unsymmetrical complexes bearing smaller *N-*alkyl groups (Figure 7) were reported by Blechert and co-workers [21].

In addition to the concept that the presence of more electron-donating alkyl groups on the NHC could lead to enhanced σ-donor properties, and, consequently, to higher catalytic activity, the authors postulated that the unsymmetrical nature of the NHC ligands could improve *E/Z* selectivity in CM reactions and diastereoselectivity in RCM reactions altering the environment of key metathesis intermediates. Complexes **24** and **25** were found to exist in solution as a single rotational isomer having the benzylidene moiety located under the mesityl group, and for complexes **24b** and **25b** this orientation was observed also in the solid state. Some metathesis reactions performed in this study with **24b** and **25b** in comparison to **GII-SIMes** and **HGII-SIMes** are summarized in Table 1. In the model RCM reaction of *N,N*-diallyl-*p*-toluenesulfonamide (**26**, Table 1, entry 1), catalysts **24a** and **24b** showed activities similar to that of **GII-SIMes**. They also exhibited different *E/Z* selectivities in CM transformations (e.g., Table 1, entry 2), and gave improved selectivities in a diastereoselective RCM reaction (Table 1, entry 3).

**Table 1:** Examples of metathesis reactions performed with catalysts **24a** and **24b**.^a^

entry	substrate	product	complex	loading (mol %)	conversion (%)

1	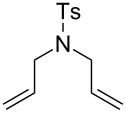 **26**	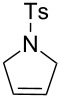 **27**	**GII-SIMes** **24a** **HGII-SIMes** **24b**	0.02 0.02 0.02 0.02	50 56 66 56
2	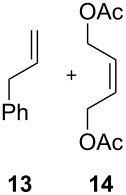	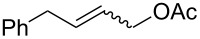 **15**	**GII-SIMes** **24a** **HGII-SIMes** **24b**	3 3 3 3	79 (*E*/*Z* = 6:1) 72 (*E*/*Z* = 3:1) 84 (*E*/*Z* = 6:1) 76 (*E*/*Z* = 6:1)
3	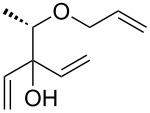 **28**	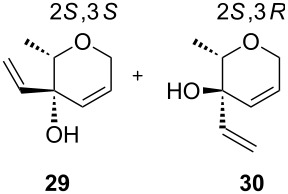	**GII-SIMes** **24a** **HGII-SIMes** **24b**	3 3 3 3	95 (**29**/**30** = 1.6:1) 92 (**29**/**30** = 1.7:1) 95 (**29**/**30** = 1.5:1) 95 (**29**/**30** = 2.0:1)

^a^Reactions performed in refluxing dichloromethane [21].

**Figure 8:**
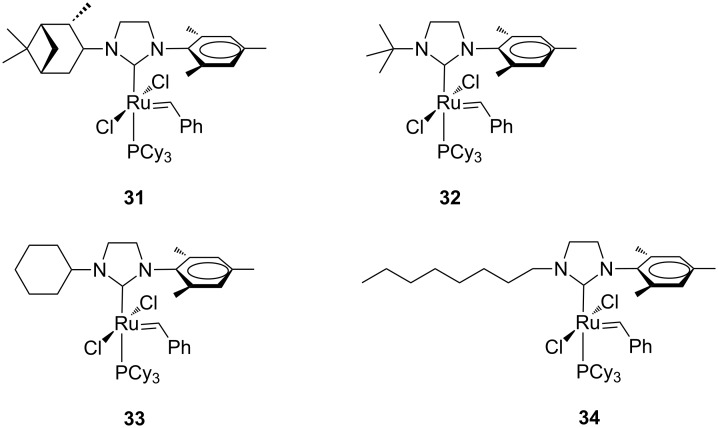
Grubbs-type complexes **31**–**34** with *N*-alkyl, *N’-*mesityl NHCs.

Ledoux, Verpoort et al. described a series of phosphine-containing unsymmetrical catalysts **31**–**34** characterized by alkyl N-substituents with variable steric bulk (Figure 8) [22].

**Figure 9:**
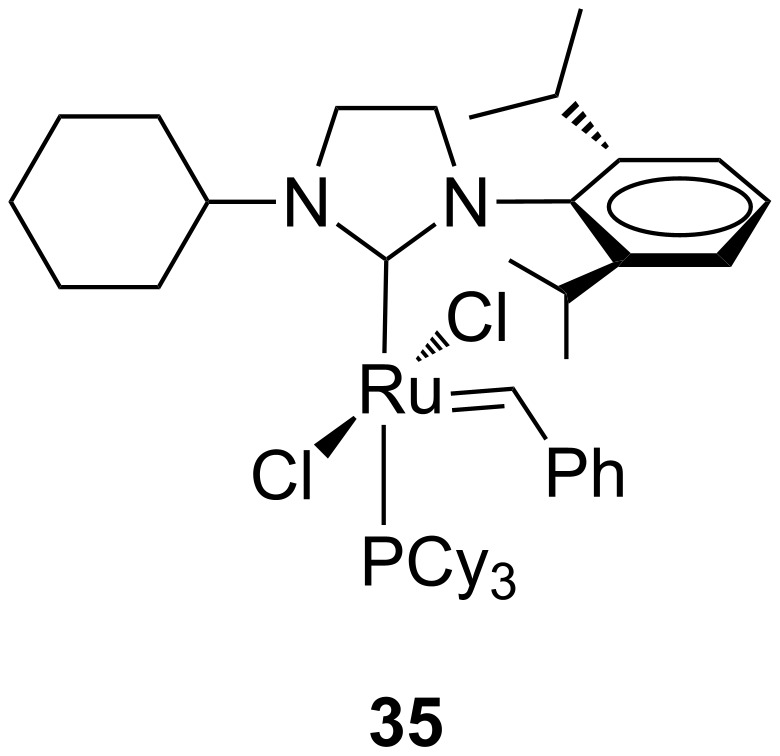
Grubbs-type complex **35** with an *N-*cyclohexyl, *N’*-2,6-diisopropylphenyl NHC.

The catalytic performances of these complexes and of complex **24a** were evaluated for the RCM of diethyl diallylmalonate (**7**) and the ROMP of *cis*-1,5-cyclooctadiene (**16**). In the RCM reaction (Scheme 1), performed at 20 °C in CD_2_Cl_2_ at a catalyst concentration of 4.52 mM and a substrate/catalyst ratio of 200 (0.5 mol % of catalyst), a strong dependence of the catalytic activities on the steric bulkiness of the *N*-alkyl substituents was observed. Indeed, an increase in the size of the alkyl group resulted in a lower catalyst activity. Indeed, complex **24a** bearing the small methyl moiety on the nitrogen, revealed as the best performing catalyst, even surpassing the parent complex **GII-SIMes**. In the ROMP reaction (Scheme 5), carried out in different solvents and monomer/catalyst ratios, the activities of complexes **31**, **33** and **34** were superior to that of the symmetrical counterpart **GII-SIMes** at low COD/catalyst loading in CDCl_3_. In general, the complexes were less dependent on the solvent used with respect to **GII-SIMes**. Catalyst **32**, having a bulky *N*-*tert*-butyl substituent on the NHC, displayed a considerably lower activity than the other tested catalysts. The replacement of the mesityl group by a 2,6-diisopropylphenyl group as in complexes **24a** and **33** led preferentially to bis(NHC)-coordinated complexes, which showed metathesis activity only at elevated temperatures [23]. However, the mono(NHC) complex **35** (Figure 9) was isolated and tested in the RCM of **7** and the ROMP of *cis*-1,5-cyclooctadiene (**16**), where it displayed a fair olefin metathesis activity compared to the benchmark catalyst **GII-SIMes** [23].

**Figure 10:**
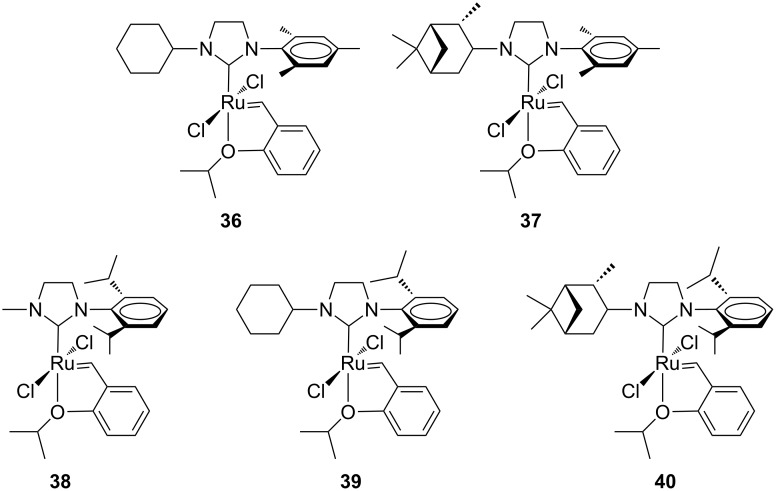
Hoveyda-type complexes with an *N*-alkyl, *N’*-mesityl (**36**, **37**) and an *N-*alkyl, *N’*-2,6-diisopropylphenyl (**38**–**40**) NHC ligand.

Studies on this class of unsymmetrical NHC ligands were also extended to the Hoveyda-type complexes **36**–**40** (Figure 10) [24]. The effect of the modified NHC ligand was investigated in model metathesis reactions (RCM of **7**, ROMP of **16** and CM of **13** with acrylonitrile) in comparison to complex **24b** and the parent complexes **GII-SIMes** and **GII-SIPr**.

No real improvement in the catalytic activity was observed in any of the tested metathesis reactions, while different *E/Z* selectivities were observed in the CM of allylbenzene (**13**) with acrylonitrile. These results underline that steric differences in *N*-alkyl NHC ligands are more important than differences in their donor capacities in determining the activity and selectivity of the corresponding catalysts.

**Figure 11:**
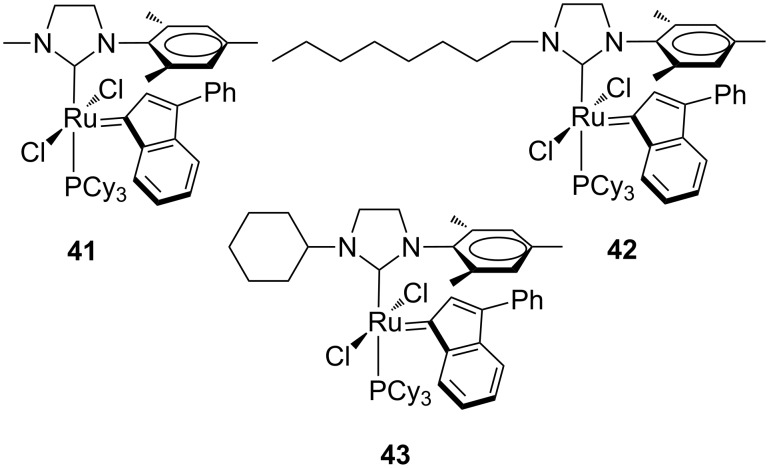
Indenylidene-type complexes **41**–**43** with *N*-alkyl, *N*’-mesityl NHCs.

Quite recently, on the basis of a previous work, Verpoort et al. reported on the synthesis and characterization of second generation ruthenium indenylidene catalysts bearing *N*-alkyl, *N’*-mesityl-substituted NHCs **41**–**43** in which the alkyl group was methyl (**41**), octyl (**42**) or cyclohexyl (**43,** Figure 11) [25].

For all of the complexes, two rotamers were observed in solution, and the most abundant species was identified as the isomer with the indenylidene moiety located under the mesityl group. Solid-state structures of the complexes showed, consistently, the same relative orientation between the indenylidene and mesityl unit. Complexes **41**–**43** were tested in various representative metathesis reactions of standard substrates and compared to the benchmark catalysts **IndII-SIMes**. Interestingly, all complexes showed a faster catalytic initiation than **IndII-SIMes**. This faster initiation may be due to the stronger *σ*-donating properties of the unsymmetrical *N*-alkyl-substituted NHC ligands. Catalyst **41** bearing the smallest-sized *N*-alkyl group on the NHC emerged as the most performing catalyst in both initiation and propagation stages, even with respect to **IndII-SIMes**. Indeed, besides its faster initiation, complex **41** offers a less encumbered NHC for the approach of substrates to the metal center during the metathesis process. The performance of complex **41** also was compared with that of the benzylidene analogue **GII-SIMes** in the RCM of **7** (Scheme 1) using various catalyst loadings (0.125–0.5 mol %). Although the benzylidene complex **GII-SIMes** exhibited a faster initiation than the indenylidene complex **41** with all the used catalyst loadings, the latter outperformed **GII-SIMes** in the overall catalyst efficiency, especially at the lowest catalyst loading of 0.125 mol %.

**Figure 12:**
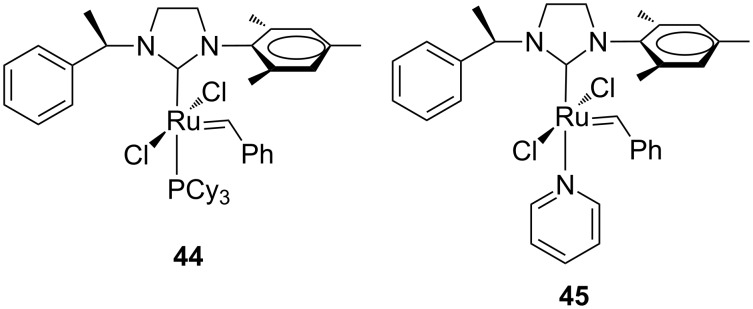
Grubbs-type complex **44** and its monopyridine derivative **45** containing a chiral uNHC.

In 2008, Blechert and Buchmeiser et al. introduced a ruthenium complex featuring an unsymmetrical, chiral NHC ligand **44** and its pyridine derivative **45** (Figure 12) [26].

**Scheme 6:**
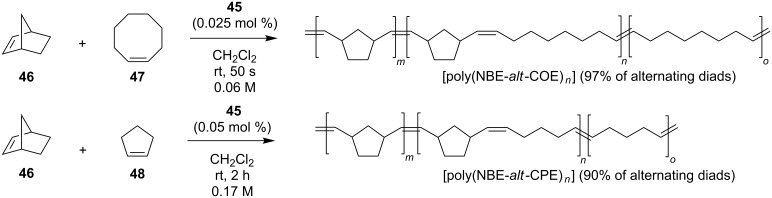
Alternating copolymerization of **46** with **47** and **48**.

Both complexes revealed as efficient systems to promote the alternating copolymerization of norbornene (NBE, **46**) with cyclooctene (COE, **47**) and cyclopentene (CPE, **48**), respectively (Scheme 6).

**Figure 13:**
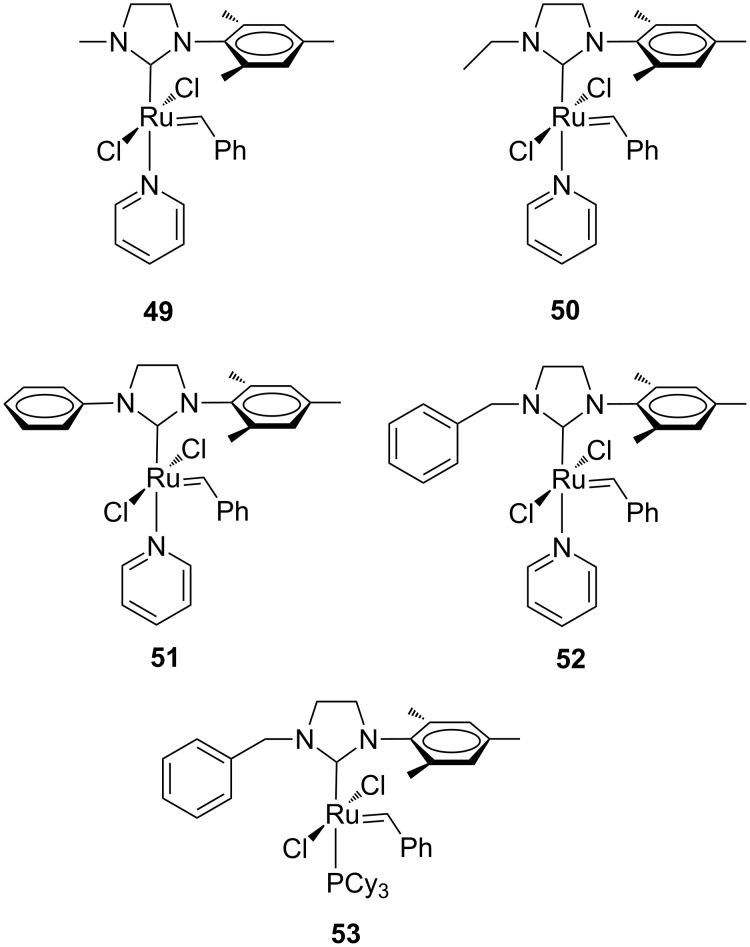
Pyridine-containing complexes **49**–**52** and Grubbs-type complex **53**.

An NBE/COE ratio of 1:50 was found necessary to realize a copolymer containing 97% of alternating diads ([poly(NBE-*alt*-COE)*_n_*]), while an NBE/CPE ratio of only 1:7 resulted in the formation of a copolymer with roughly 90% of alternating diads ([poly(NBE-*alt*-CPE)*_n_*]), representing the highest value found until then. The selectivity in the copolymerization was mainly ascribed to the steric interaction between the 2-phenylethyl substituent at the nitrogen and the growing polymer chain. This study was then extended to a series of unsymmetrical pyridine-containing Ru benzylidenes (Figure 13) with *N*-alkyl (**49**, **50**), *N*-phenyl (**51**) and *N*-benzyl (**52**) substituents in comparison to their parent phosphine-containing catalysts **24a**, **25a**, **1a** and **53** [27].

**Figure 14:**
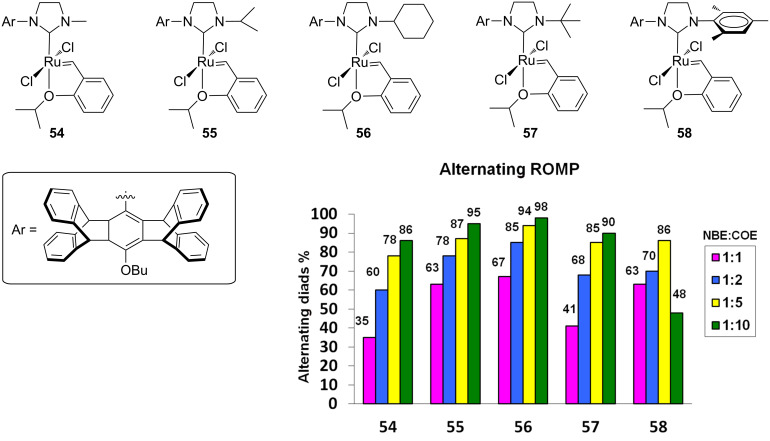
Hoveyda-type complexes **54**–**58** in the alternating ROMP of NBE (**46**) and COE (**47**).

Complexes **49** and **52** were obtained as monopyridine adducts, while complexes **50** and **51** were obtained as a mixture of mono- and bis(pyridine) adducts. In terms of initiation efficiency, the pyridine-derivatives turned out to be more efficient than the corresponding phosphine-containing complexes. In the copolymerization of NBE (**46**) and COE (**47**), complexes **49**–**52** afforded the corresponding copolymers with 95–97% of alternating diads and high *cis* content. In the copolymerization of NBE (**46**) and CPE (**48**), copolymers with 79–91% of alternating diads were obtained. More recently, Plenio and co-workers described a new class of Hoveyda–Grubbs-type catalysts with an *N*-alkyl, *N’*-pentiptycenyl NHC ligand (**54**–**57**, Figure 14). The complex **58** having an *N*-mesityl, *N’*-pentiptycenyl NHC was also reported [28].

These complexes disclosed an excellent degree of alternation in the copolymerization of NBE and COE (0.05 mol % of catalyst, [NBE] = 0.14 M). Especially catalyst **56** having a cyclohexyl N-substituent provided the copolymer with the highest amount of alternating diads (98%) at an NBE/COE ratio of 1:10. However, the molecular mass of the copolymers was far lower than the theoretical value, suggesting that competitive chain-termination reactions occur. The pronounced steric bulk on the pentiptycenyl side of the NHC ligand compared to the other less hindered side determines two differently accessible active sites around the metal and different rates of monomer incorporation, thus dominating the selectivity in the formation of alternating copolymers. The nature of the alkyl group also plays a role in the formation of alternating diads. Indeed, the proportion of alternating copolymer increases moving from the small methyl group (**54**) to the large cyclohexyl group (**56**).

**Figure 15:**
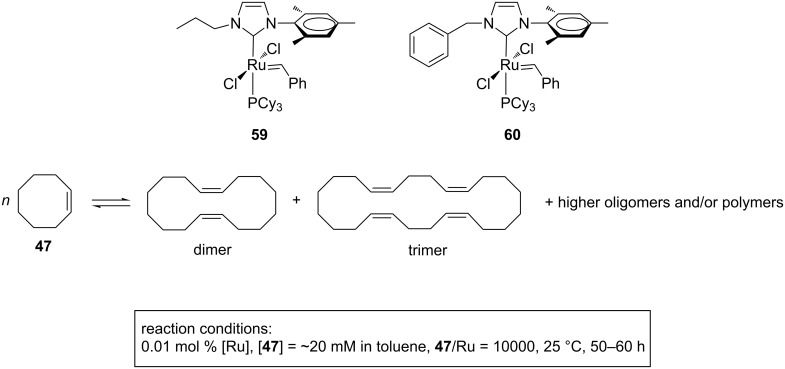
Catalysts **59** and **60** in the tandem RO–RCM of **47**.

Unsymmetrical catalysts based on NHC units possessing one alkyl substituent (propyl (**59**) or benzyl (**60**)) and one mesityl substituent (Figure 15) at the nitrogen atoms were investigated by Copéret and Thieuleux et al. in the tandem ring-opening–ring-closing alkene metathesis (RO–RCM) of *cis*-cyclooctene (**47**) and their performance were compared to those of the classical **GII-SIMes** and **GII-IMes** [29].

The dissymmetry of the NHC ligand in **59** and **60** allowed for the selective formation of cyclic dimeric and trimeric products in place of polymers from cyclooctene, while the symmetrical analogues **GII-SIMes** and **GII-IMes** led mainly to polymers (Figure 15).

**Figure 16:**
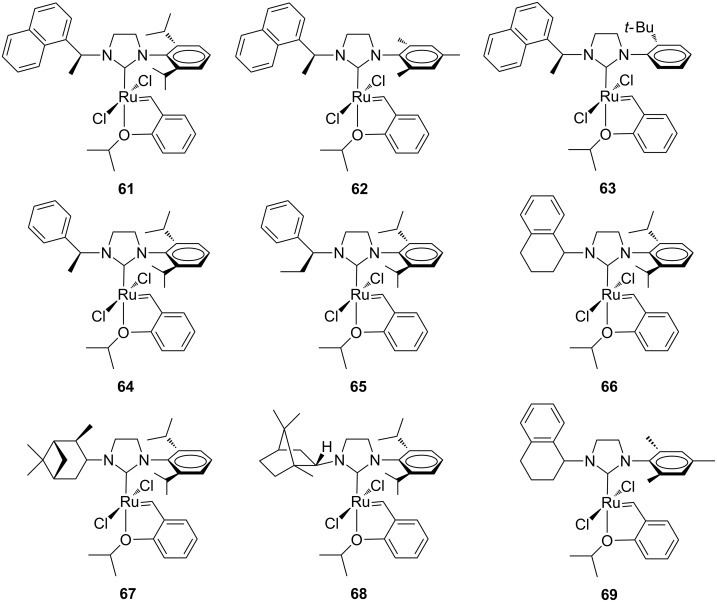
Hoveyda-type complexes **61**–**69** with *N*-alkyl, *N’-*aryl NHCs.

**Scheme 7:**
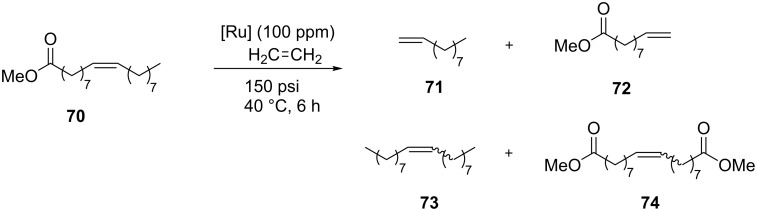
Ethenolysis of methyl oleate (**70**).

Following a study on degenerate metathesis reactions that had highlighted a strong catalytic preference of unsymmetrical *N*-alkyl, *N*’-aryl complexes to propagate as a methylidene species [30], Grubbs and co-workers developed a variety of unsymmetrical metathesis Hoveyda-type complexes (**61**–**69**, Figure 16) for applications in the ethenolysis of methyl oleate (**70**, Scheme 7) [31].

The ethenolysis reaction, in fact, requires catalyst stability as a propagating methylidene species to achieve high product selectivity and turnover numbers (TONs). The catalysts **61**–**69**, tested together to the phosphine-containing catalyst **32**, were found to be highly selective toward the formation of the desired ethenolysis products **71** and **72** (Scheme 7), and provided good yields and TONs at 50 °C and low catalyst loading (100 ppm, Table 2). Furthermore, many of the screened catalysts showed good stability toward propagation as a methylidene species. The observed selectivity seems to be controlled by the NHC sterics, as increasing steric bulkiness of the NHC ligand leads to greater selectivity and improves stability.

**Table 2:** Ethenolysis of methyl oleate (**70**) with catalysts **61**–**69**.

entry	complex	conversion (%)	selectivity (%)	yield (%)	TON

1	**61**	54	86	46	4620
2	**62**	11	77	9	845
3	**64**	52	86	45	4450
4	**65**	42	86	36	3600
5	**66**	59	87	51	5070
6	**67**	52	89	46	4604
7	**68**	15	95	15	1460
8	**69**	17	69	11	1120

**Scheme 8:**
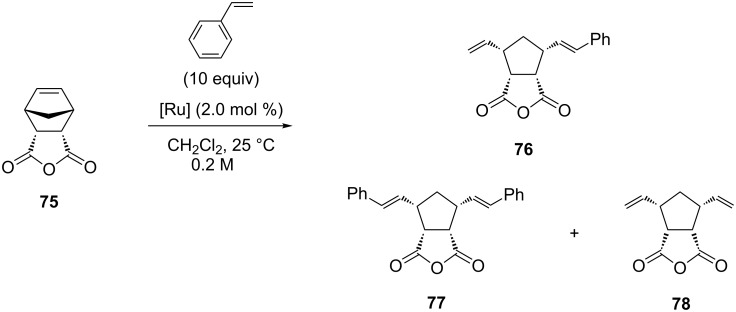
AROCM of *cis*-5-norbornene-*endo*-2,3-dicarboxylic anhydride (**75**) with styrene.

Catalyst **68** gave the highest selectivity (95%) toward terminal olefins observed until then for NHC–Ru complexes (Table 2, entry 7), but with 46% yield at 500 ppm of catalyst loading. The chiral catalysts **61**, **64**, **65**, **67** and **68** (Figure 16) were also investigated in the model asymmetric ring-opening cross metathesis (AROCM) of *cis*-5-norbornene-*endo*-2,3-dicarboxylic anhydride (**75**) with styrene (Scheme 8, Table 3) [32].

**Table 3:** AROCM of *cis*-5-norbornene-*endo*-2,3-dicarboxylic anhydride (**75**) with catalysts **61**, **64**, **65**, **67** and **68**.

entry	complex	time (h)	conversion (%)	yield (%)	ee **76** (%)

1	**61**	5.5	60	60	69
2	**64**	0.5	99	69	14
3	**65**	0.5	99	73	9
4	**67**	5.5	98	65	33
5	**68**	10.5	98	54	82

In this reaction complex **68** showed the highest selectivity for the formation of the desired product **76** (82% ee, Table 3, entry 5), comparable to the best ruthenium catalysts investigated in this AROCM reaction. All complexes gave side products **77** and/or **78** resulting from metathesis reactions of propagating ruthenium methylidene species.

**Figure 17:**
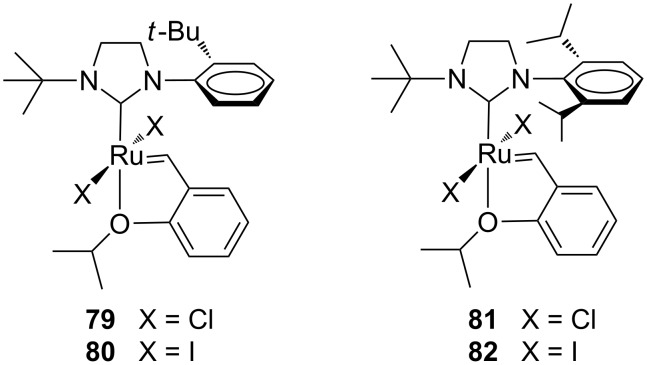
Hoveyda-type catalysts **79**–**82** with *N*-*tert*-butyl, *N’*-aryl NHCs.

In the same year, Grubbs and co-workers reported on the synthesis of highly thermally stable complexes containing a sterically encumbered *N-tert*-butyl substituent (**79**–**82**, Figure 17) which enables their application for latent olefin metathesis [33].

**Scheme 9:**
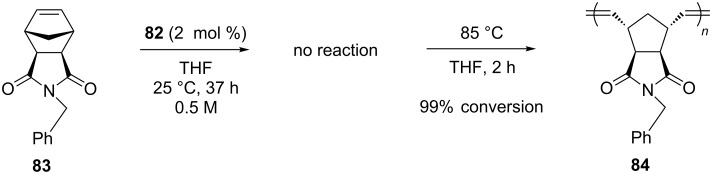
Latent ROMP of **83** with catalyst **82**.

The complexes **79** and **81** having chloride ligands exhibited excellent latent behavior toward self-CM of 1-hexene, giving no conversion at room temperature and dimerization at 85 °C. Exchanging the chloride ligands for iodide ligands led to catalysts **80** and **82** with superior latent behavior that allowed for the latent ROMP of norbornene derivatives (e.g., **83**, Scheme 9).

**Figure 18:**
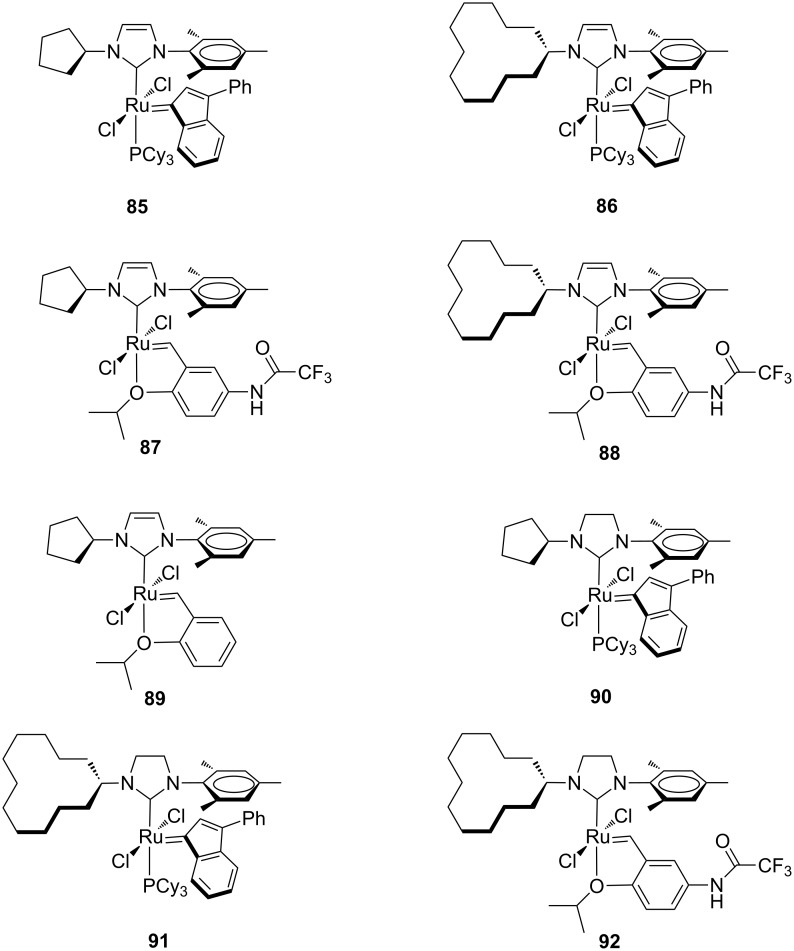
Indenylidene and Hoveyda-type complexes **85**–**92** with *N*-cycloalkyl, *N’*-mesityl NHCs.

In order to improve the selectivities in olefin metathesis, a small library of indenylidene and Hoveyda-type complexes bearing unsaturated unsymmetrical NHCs combining a flexible cycloalkyl moiety and a mesityl unit as N-substituents (**85**–**89**, Figure 18) was synthesized by Mauduit and co-workers [34]. These systems were tested in the RCM of sterically demanding diethyl allylmethallylmalonate (**9**) under standard conditions (Scheme 2) and compared to their unsymmetrical saturated NHC–Ru complexes **90**–**92** (Figure 18) as well as a set of commercially available catalysts having symmetrical IMes or SIMes NHC ligands.

**Scheme 10:**
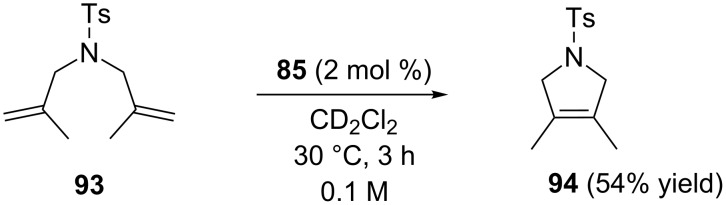
RCM of *N,N*-dimethallyl-*N*-tosylamide (**93**) with catalyst **85**.

The unsaturated indenylidene catalysts **85** and **86** were found to be more active than their saturated homologues, giving full conversions within 6 h and 24 h, respectively, thus showing better performances than **IndII-IMes** and Hoveyda-type catalysts **87**–**89**, **92**. As for the latter ones, the introduction of unsaturated NHCs with an *N*-cycloalkyl moiety did not provide any beneficial effect, since they were less efficient also than their symmetrical IMes and SIMes counterparts. The catalytic potential of the most active complex **85** with a cyclopentyl fragment on the NHC was explored in several RCM and CM reactions. Interestingly, in the RCM of *N,N*-dimethallyl-*N*-tosylamide (**93**) only 2 mol % of **85** were required to produce 54% of the tetrasubstituted tosylamide **94** within 3 h (Scheme 10).

**Scheme 11:**
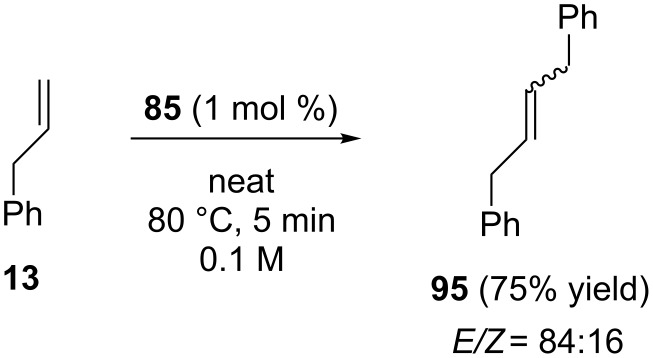
Self metathesis of **13** with catalyst **85**.

Moreover, catalyst **85** was quite efficient under neat conditions for the self metathesis of allylbenzene (**13**), showing no trace of isomerized byproducts (Scheme 11).

More recently, Olivier-Bourbigou and Mauduit demonstrated the ability of unsymmetrical *N*-cycloalkyl Ru–indenylidene catalysts for the selective self metathesis of linear α-olefins to longer internal linear olefins in the absence of additives to prevent isomerization [35]. Catalyst **91** with a saturated NHC ligand containing a N*-*substituted cyclododecyl side chain was first evaluated at 50 ppm loading in the self metathesis of 1-octene (**96**), at 50 °C under neat conditions, in comparison to symmetrical benchmark second-generation ruthenium catalysts **IndII-SIMes**, **IndII-IMes**, **GII-SIMes** and **HGII-SIMes** (Table 4). Complex **91** was found to give 70% conversion of 1-octene (**96**) to the desired 7-tetradecene (**97**) with high selectivity (98% after 1 h, Table 4, entry 1). Moreover, the selectivity did not change over time (Table 4, entry 2). A lower selectivity was observed with **IndII-SIMes** (Table 4, entries 3 and 4 ) and **GII-SIMes** (Table 4, entry 5), while **IndII-IMes** was inactive (Table 4, entry 6) and **HGIIMes** gave only low conversion (Table 4, entry 7).

**Table 4:** Self metathesis of 1-octene (**96**).

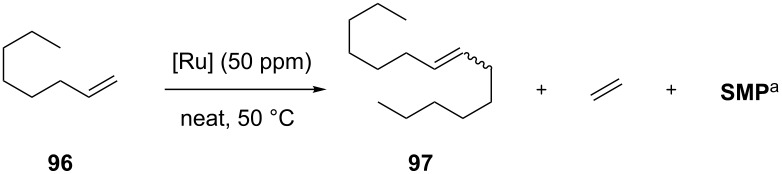				

entry	complex	time (h)	conversion (%)	selectivity (%)

1	**91**	1	70	98
2	**91**	4	70	98
3	**IndII-SIMes**	1	45	94
4	**IndII-SIMes**	2	76	80
5	**GII-SIMes**	2	80	85
6	**IndII-IMes**	4	<1	–
7	**HGII-SIMes**	4	30	98
8	**85**	2	59	99
9	**86**	4	55	98

^a^SMP: secondary metathesis products (mixture of C_3_–C_13_ olefins) [35].

To render this process really attractive for industrial application, the authors also evaluated the lower-cost catalysts **85** and **86** in the self metathesis of **96** (Table 4, entries 8 and 9, respectively). Indeed, the one-step multicomponent synthesis of unsaturated unsymmetrical NHCs could provide a cost-effective alternative to the multistep synthesis of their saturated counterparts [36]. The catalyst **85** was identified as the catalyst of choice for the selective metathesis of linear α-olefins and was successfully applied to selectively re-equilibrate the naphtha fraction (C_5_–C_8_) of a Fischer–Tropsch feed derived from biomass to higher value added olefins (C_9_–C_14_) that can serve as plasticizer and detergent precursors. An excellent olefin distribution with no isomerization was observed without the use of any additive even after 24 h of reaction performed at 50 °C under neat conditions.

#### *N*-Alkyl substituents possessing functionalities or heteroatoms

**Figure 19:**
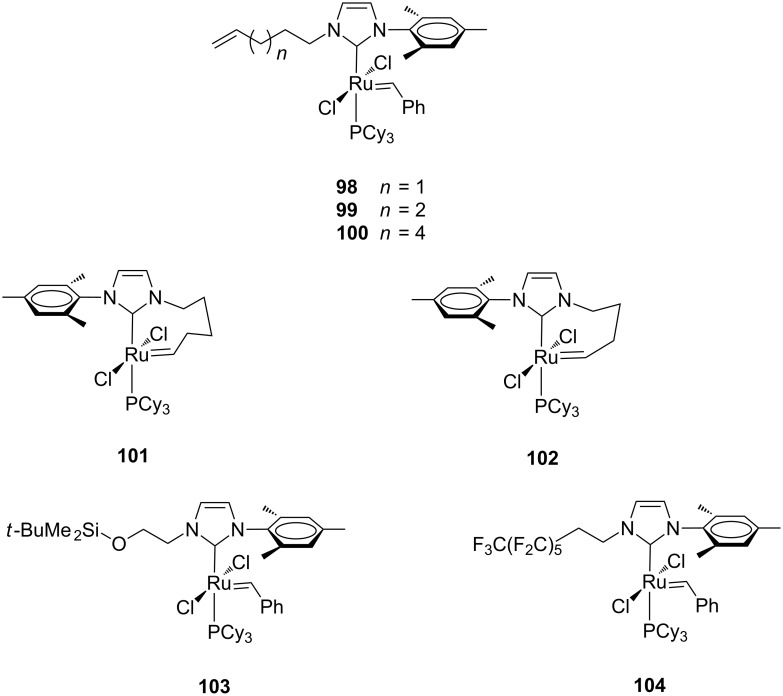
Grubbs-type complexes **98**–**104** with *N*-alkyl, *N’*-mesityl NHCs.

In 2001, the Fürstner group reported on phosphine-containing ruthenium complexes having unsymmetrical NHCs characterized by an alkenyl chain replacing one of the *N*-mesityl groups of the NHC ligand (**98**–**100**, Figure 19) [37]. The complexes **98**–**100** were able to metathesize their own ancillary ligands, thus leading to species in which the NHC ligand is bound to the Ru=CHR moiety to form a metallacycle (**101** and **102**, Figure 19). The basic idea was that these catalysts might be able to regenerate themselves upon consumption of the monomer in the reaction media. Variants of these complexes with a silyl ether or a perfluoroalkyl chain on one of the nitrogens of the NHC were also presented (**103** and **104**, Figure 19).

The catalytic behavior of complexes **98**–**100** and **101**, **102** was tested in the RCM of *N*,*N*-dimethallyl-*N*-tosylamide (**93**) to form the corresponding tetrasubstituted cycloolefin **94** (Scheme 10; reaction performed in toluene at 80 °C with 5 mol % of catalyst). All the complexes were able to achieve the cyclization, although the catalytic activity of the homologous series **98**–**100** was found to be strongly dependent on the tether length between the alkene group and the metal center. This effect is likely related to their different ability in forming the corresponding chelate complexes in situ (Figure 19).

**Figure 20:**
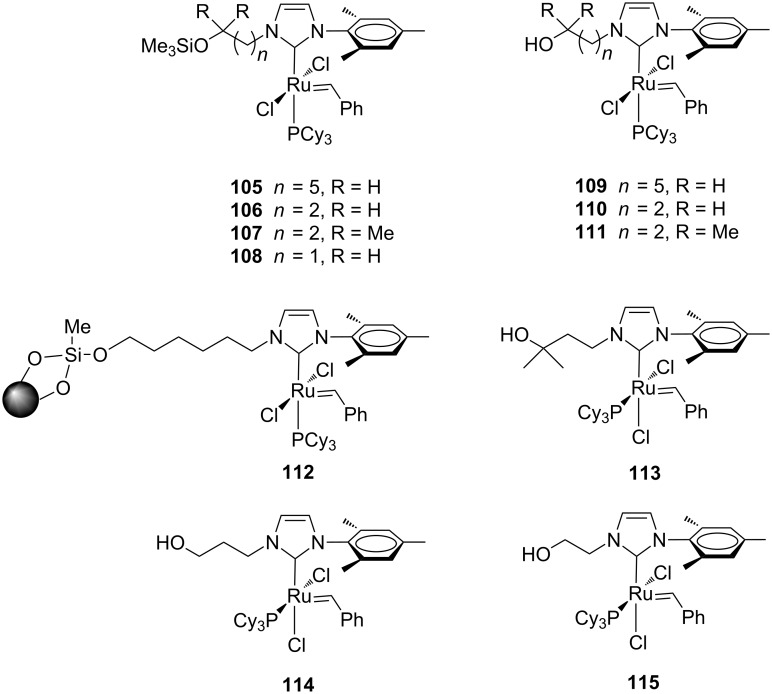
Grubbs-type complexes **105**–**115** with *N*-alkyl, *N’*-mesityl ligands.

Importantly, later on Grubbs and co-workers utilized this kind of catalysts, featuring a chelating N-to-Ru arm, for the preparation of cyclic polymers from cyclic monomers via a ring-expansion metathesis polymerization (REMP) process [38–39]. With the aim of developing catalysts suitable for covalent immobilization on various supports, Fürstner et al. reported on the preparation of some unsymmetrical complexes containing pendant protected (**105**–**108**) and unprotected (**109**–**111**) hydroxyalkyl chains on their NHCs (Figure 20) [40].

Complex **109** was easily immobilized on functionalized silica gel and the resulting complex **112** (Figure 20) was tested in prototype RCM reactions. In comparison to its homogeneously soluble analogues **109** and **110**, complex **112** required longer reaction times to give the same yields, but was reusable up to three times.

Interestingly, during investigations carried out to anchor this type of ruthenium complexes by physisorption rather than chemisorption, an unexpected molecular rearrangement of their ligand sphere, determining a *cis* orientation of the neutral ligands, was observed (**113** and **114**, Figure 20). The same unusual *cis* configuration was displayed by complex **115** (Figure 20) upon release from its precursor **108** by deprotection under acidic conditions.

The *cis* isomers **113**–**115** exhibited catalytic activity only at high temperatures, where they likely reassume the *trans* form which is characteristic for the Grubbs-type ruthenium carbene complexes.

**Figure 21:**
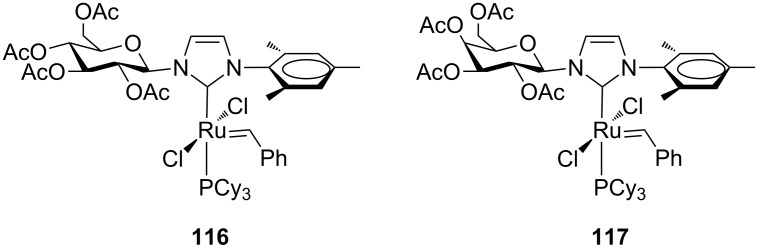
Complexes **116** and **117** bearing a carbohydrate-based NHC.

In order to develop a new structural class of highly performing NHC-based metathesis catalysts with *N*-alkyl groups, ruthenium benzylidene complexes containing carbohydrate-based NHCs derived from glucose (**116**) and galactose (**117,** Figure 21) were reported in 2009 [41].

These complexes were characterized in solution by NMR techniques which revealed, at room temperature, the presence of rotameric species resulting from rotation about the Ru–C(benzylidene) bond. The catalytic behavior of **116** and **117** was examined in standard RCM, CM, ROMP olefin metathesis reactions. Interestingly, **116** and **117** differing only at one stereocenter showed different kinetic behavior in the RCM of diethyl diallylmalonate (**7,** Scheme 1; reaction temperature 40 °C), where **117** displayed a higher activity than catalyst **116**. Furthermore, they showed surprising selectivity (*E/Z* ratio around 3) in the CM of allylbenzene (**13**) and *cis*-1,4-diacetoxy-2-butene (Scheme 4; reaction temperature 40 °C) compared to the benchmark catalysts **GII-IMes** and **GII-SIMes**, indicating that the steric bulk of the carbohydrate plays a role in influencing the geometry of the resulting olefinic product. Given the chiral nature of the carbohydrate attached to the NHC, complexes **116** and **117** were tested in the AROCM of a variety of norbornene derivatives with styrene. While isolated yields were generally excellent, enantiomeric excesses were poor.

**Figure 22:**
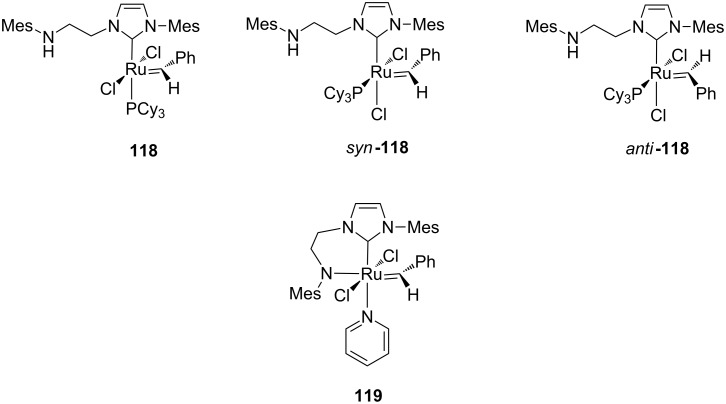
Complexes **118** and **119** bearing a hemilabile amino-tethered NHC.

The effect of a dangling amine tether incorporated into the NHC ligand on the catalytic efficiency of ruthenium benzylidene complexes was examined by Fryzuk et al. (**118**, Figure 22) [42].

NMR studies showed that complex **118** exists as a mixture of two rotational isomers in a 7:1 ratio. The major isomer was characterized by X-ray crystallography, while the minor isomer was characterized only in solution and was identified as consistent with two possible structures (*syn-* and *anti*-**118**). In *syn*-**118** the two chloro ligands are *cis* disposed and the PCy_3_ unit is *cis* to both the NHC and the benzylidene, whereas in *anti*-**118** the PCy_3_ unit and the benzylidene are *trans* with respect to the Ru=CHPh double bond. Moreover, no coordination of the tethered amine to the ruthenium center was detected in the species **118** by NMR spectroscopy. Evidence for coordination of the amino arm in solution and in the solid state was observed in its derived monopyridine adduct **119** (Figure 22). Complex **118** was found less active than **GII-SIMes** and **GII-IMes** in model RCM of **7** and ROMP of **16** (see Scheme 1 and Scheme 5, respectively). In the RCM of **7**, catalyst **118** gave 25% conversion in 30 min, while **GII-SIMes** and **GII-IMes** reached 96% and 74% conversion, respectively, within the same time. As for the ROMP of **16**, only 40% conversion was observed after 4 h with **118**, while full conversion was registered for **GII-SIMes** and **GII-IMes** in 6 and 80 min, respectively. The catalyst efficiency is further reduced in the pyridine derivative **119**, suggesting that the pendant amine is deleterious for catalyst performance.

### Ruthenium catalysts coordinated with *N-*benzyl, *N’*-aryl NHCs

**Figure 23:**
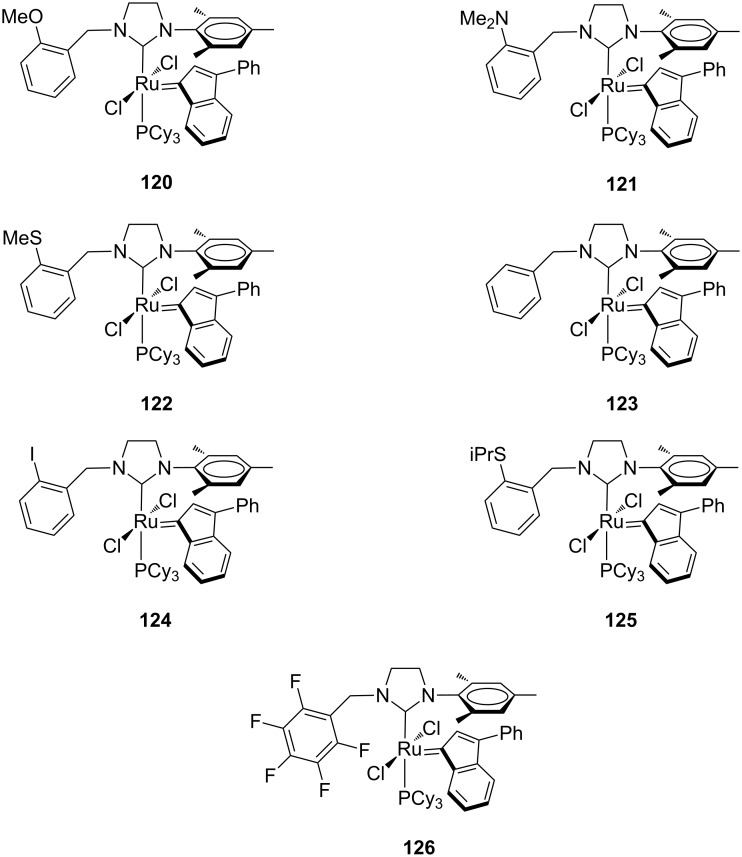
Indenylidene-type complexes **120**–**126** with *N*-benzyl, *N’*-mesityl NHCs.

The effect of replacing one of the mesityl groups of the NHC ligand with a flexible benzyl group on the catalytic properties of the resulting ruthenium complexes was studied by Grela and co-workers, who synthesized indenylidene complexes **120**–**126** [43–44] (Figure 23). Substituents in the benzyl group were introduced to modify the steric and electronic properties of the ligand and/or to allow additional coordination to the metal center.

The catalytic behavior of **120**–**126** was investigated in standard metathesis reactions using commercial grade solvents in air and compared to that of commercially available **IndII-SIMes.** Catalysts **120**, **121**, **123**, **124** and **126** showed a better performance than **IndII-SIMes** in the RCM of **7** (Scheme 1), whereas the sulfur-containing catalysts **122** and **125** displayed lower activity. In more detail, **120**, **121**, **123** and **124** exhibited similar behavior, in spite of the different nature of aryl substituents, while **126** was found to be less efficient. Solvent tests on **IndII-SIMes**, **123** and **126** demonstrated that dichloromethane is a better solvent with respect to toluene, even if in toluene the initiation of catalyst **126** is faster. The low activity of **122**, **125** and **126** was rationalized by supposing the presence of an interaction between the metal and the heteroatoms of the benzyl substituents [15,43–44]. Complexes **120**, **121**, **123**, and **124** significantly outperformed commercial **IndII-SIMes** in the RCM of diethyl allylmethallylmalonate (**9**) as well. On the contrary, they appeared not suitable in the synthesis of tetrasubstituted olefins. Indeed, they were tested at 60 °C in the RCM of *N,N-*dimethallyl-*N-*tosylamide (**93**, Scheme 10; reaction performed in toluene at 80 °C with 5 mol % of the catalyst), giving conversions between 30–40%, as observed also for the commercial catalyst **IndII-SIMes**.

The catalysts **120** and **121** were also tested in the ring-closing ene–yne metathesis reaction (RCEYM) of standard substrate **127**. Both catalysts revealed slightly more active than **IndII-SIMes**, with **121** being the most efficient (Table 5, entry 1). Catalyst **120** showed the highest activity in the RCM of the amide-based substrate **129** (Table 5, entry 2) and in the CM of **13** with **14**, but with a slightly lower *Z*-selectivity (Table 5, entry 3).

**Table 5:** Metathesis reactions of standard substrates.

entry	substrate	product	catalyst (mol %)	*T* (°C)	*t* (h)	isolated yield (%)

1	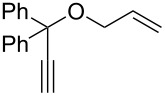 **127**	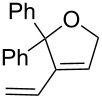 **128**	**IndII-SIMes** (2) **120** (2) **121** (2) **134** (2) **135** (2) **136** (2) **137** (2) **138** (2) **139** (2) **140** (2) **141** (2) **142** (2)	30 30 30 40 40 50 50 50 50 50 50 50	8 6 5 8 8 2 2 2 1.5 1.5 1.5 1.5	96^a^ 94^a^ 96^a^ 99^b^ 99^b^ 92^c^ 91^c^ 92^c^ 89^c^ 91^c^ 89^c^ 91^c^
2	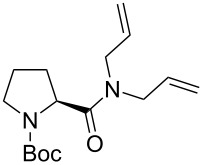 **129**	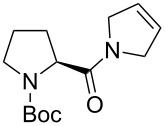 **130**	**IndII-SIMes** (1) **120** (1) **121** (1) **136** (1) **137** (1) **138** (1) **139** (1) **140** (1) **141** (1) **142** (1)	50 50 50 50 50 50 50 50 50 50	2.5 1 2 1.25 2 1 3 3 3 3	94^a^ 96^a^ 91^a^ 87^c^ 89^c^ 92^c^ 85^c^ 94^c^ 88^c^ 90^c^
3	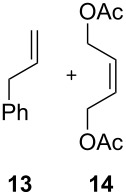	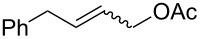 **15**	**IndII-SIMes** (2.5) **120** (2.5) **121** (2.5) **134** (2.5) **135** (2.5) **136** (2.5) **137** (2.5) **138** (2.5) **139** (2.5) **140** (2.5) **141** (2.5) **142** (2.5)	30 30 30 30 30 50 50 50 50 50 50 50	20 20 20 20 20 2 2 2 2 1.5 1.5 1.5	74 (*E*/*Z* = 8:1)^a^ 80 (*E*/*Z* = 9:1)^a^ 74 (*E*/*Z* = 11:1)^a^ 45 (*E*/*Z* = 4:1)^b^ 86 (*E*/*Z* = 5:1)^b^ 89 (*E*/*Z* = 7.1:1)^c^ 76 (*E*/*Z* = 7.9:1)^c^ 93 (*E*/*Z* = 6:1)^c^ 74 (*E*/*Z* = 3.6:1)^c^ 80 (*E*/*Z* = 7:1)^c^ 81 (*E*/*Z* = 8:1)^c^ 78 (*E*/*Z* = 6.5:1)^c^

^a^Ref [43]; ^b^Ref [45]; ^c^Ref [46].

**Scheme 12:**
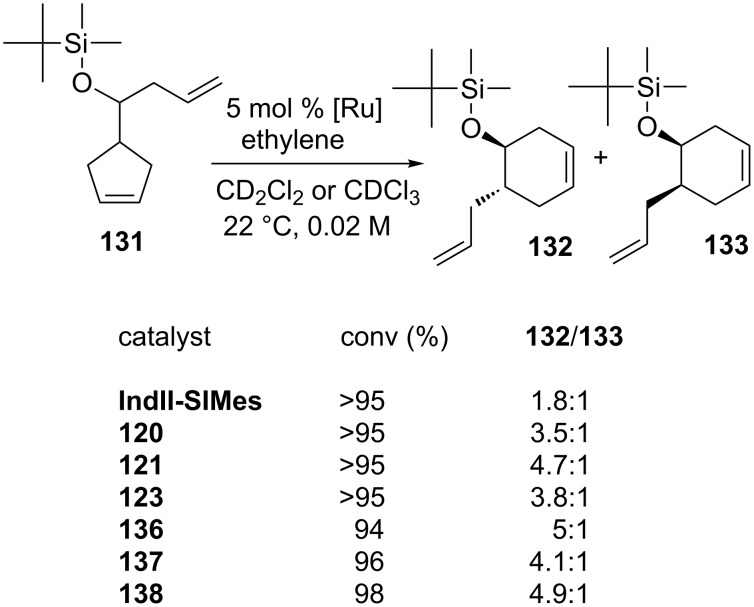
Diastereoselective ring-rearrangement metathesis (dRRM) of cyclopentene **131**.

Finally, in the presence of catalysts **120**, **121** and **123**, diastereoselectivities higher than those achieved in the presence of **GII-SIMes**, **HGII-SIMes** and **IndII-SIMes** were observed in the diastereoselective ring-rearrangement metathesis (dRRM) of cyclopentene **131** (Scheme 12).

**Figure 24:**
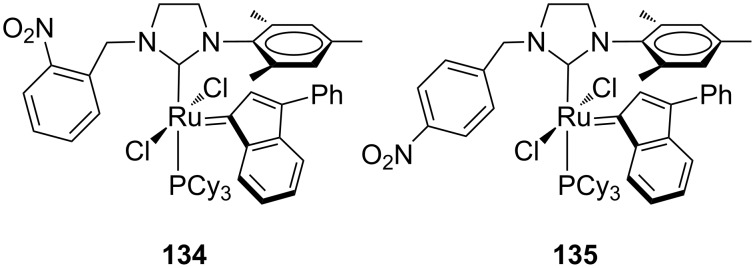
Indenylidene-type complexes **134** and **135** with *N*-nitrobenzyl, *N*’-mesityl NHCs.

The presence of a nitro group at the *ortho* or *para* positions of the benzyl substituent (**134** and **135** in Figure 24), reported by Malinowska and co-workers [45], led to higher activities in the RCM of **7** and **9** (Schemes 1 and 2), with respect to the commercial **IndII-SIMes**, but significantly lower if compared to catalysts **120**, **121**, **123** and **124**. A scarce activity toward the formation of tetrasubstituted olefin **12** (Scheme 3) was also observed. Complexes **134** and **135** were tested in RCEYM of **127** (Table 5, entry 1) showing a good efficiency and in the CM of **13** and **14** (Table 5, entry 3), where interesting *Z*-selectivities can be achieved.

**Figure 25:**
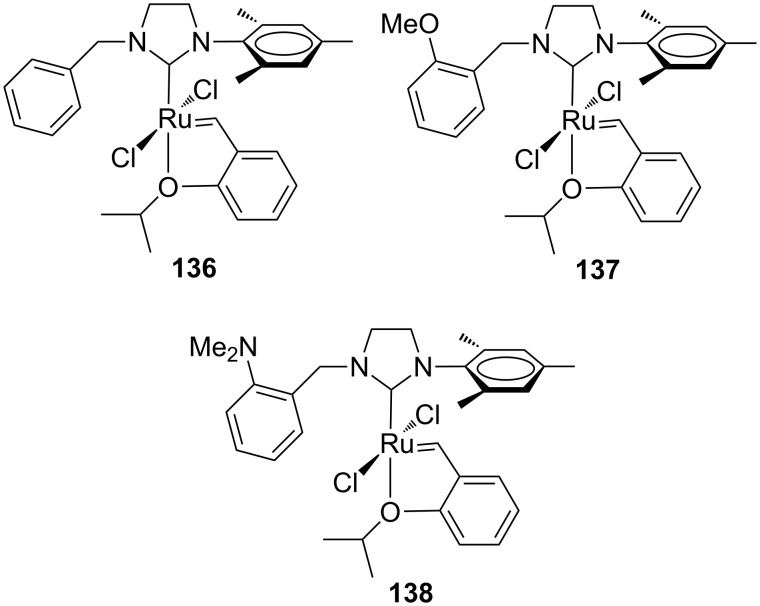
Hoveyda-type complexes **136–138** with *N*-benzyl, *N’*-mesityl NHCs.

**Figure 26:**
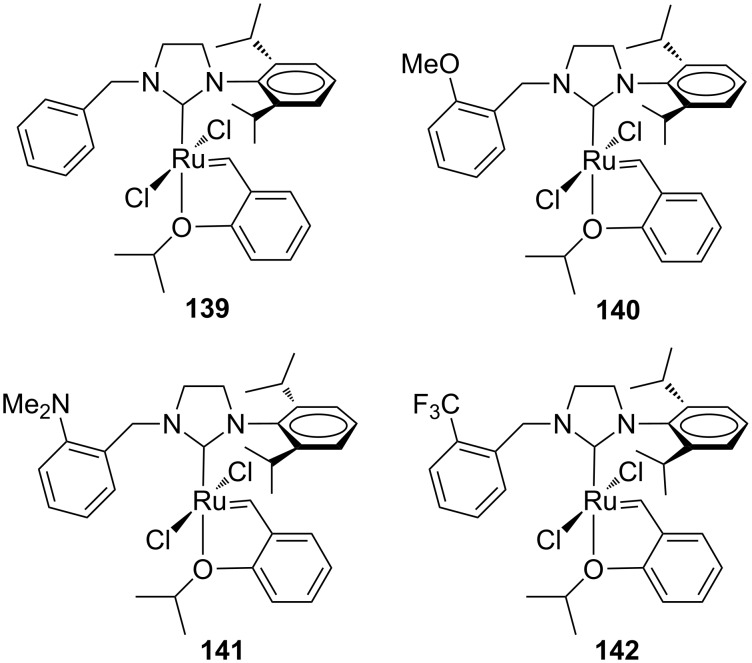
Hoveyda-type complexes **139**–**142** with *N*-benzyl, *N’*-Dipp NHC.

Recently, Grela and co-workers modified the previously reported *N*-benzyl, *N’*-aryl NHC–Ru complexes **120**, **121** and **123**, by synthesizing the analogous Hoveyda-type derivatives **136**–**138** (Figure 25). Additionally, the behavior of catalysts **136**–**138** was compared with that of complexes bearing an *N*-Dipp (Dipp = 2,6-diisopropylphenyl) substituent in place of the *N*-mesityl group (**139**–**142** in Figure 26) [46].

As expected, the *N’*-Dipp complexes displayed a higher stability with respect to the *N’*-mesityl complexes. Nevertheless, complexes **136**–**138** were more active than **139**–**142** in the RCM of **7**, conducted at 50 °C and none of those catalysts outperformed **HGII-SIMes** and **HGII-SIPr**. Analogous results were observed in the RCM of more crowded substrates. The similar behavior of **141** and **142** indicated that steric effects are more relevant than electronic effects.

Catalysts **136**–**142** were tested in the RCEYM of **127**, in the RCM of **129** and in the CM of **13** and **14** (Table 5, entry 3). According to the experimental results, mesityl-bearing catalysts generally gave better yields than Dipp-containing analogues. In the presence of **136**–**138**, a high selectivity in the dRRM of cyclopentene **131** was also observed (Scheme 12). Self metathesis of 1-octene (**96**) was conducted in the presence of **136**, **137**, **139** and **140**, in order to selectively obtain tetradec-7-ene (**97**). The presence of the *N*-benzyl substituent was crucial to achieve high yield (up to 80%) of the desired product, whereas commercial **HGII-SIMes** and **HGII-SIPr**, despite the higher reaction rate, gave mainly a mixture of byproducts.

### Ruthenium catalysts coordinated with *N*-heteroarylmethyl, *N*’-aryl NHCs

**Figure 27:**
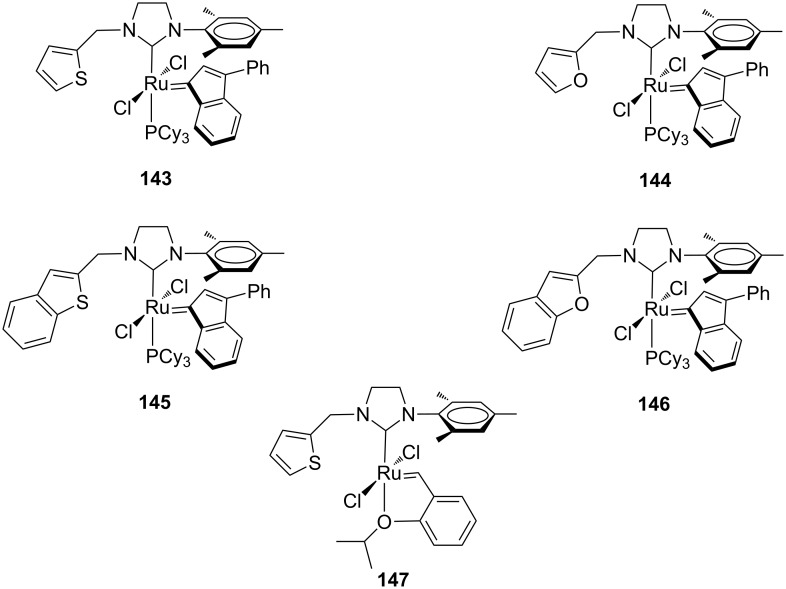
Indenylidene (**143**–**146**) and Hoveyda-type (**147**) complexes with *N*-heteroarylmethyl, *N’*-mesityl NHCs.

To further modify the electronic and steric properties of the NHC ligand and consequently, to improve efficiency of the resulting ruthenium catalysts, the Grela group focused on the development of new ruthenium indenylidene and Hoveyda-type complexes bearing unsymmetrical NHCs containing a heteroaromatic moiety (**143**–**147**, Figure 27) [47].

**Figure 28:**
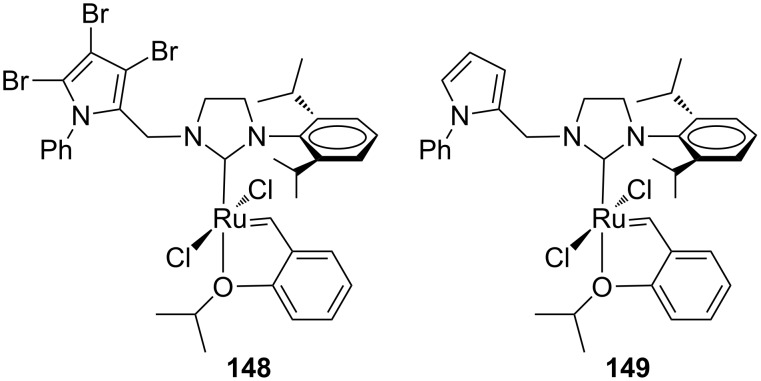
Hoveyda-type complexes **148** and **149** with *N*-phenylpyrrole, *N’*-mesityl NHCs.

The catalytic performances of **143**–**147** were examined in model RCM and CM metathesis reactions under air in commercial grade toluene and compared to benchmark complexes **IndII-SIMes** and **HGII-SIMes**. Under these conditions all the catalysts tested showed very high activity in RCM transformations, with the newly developed systems requiring shorter reaction times to give quantitative conversion. In the RCEYM of **127**, complexes **143**, **146** and **147** were performing less effectively than all the other ones, however, no clear relationship between heterocyclic substituents and activity can be found. In the CM of allylbenzene (**13**) and *cis*-1,4-diacetoxy-2-butene (**14**), all of the new catalysts gave higher amounts of the *Z* isomer than **IndII-SIMes** and **HGII-SIMes**. Indeed, **143**–**147** showed *E/Z* ratios in the range of 3.2–4.0, while **IndII-SIMes** and **HGII-SIMes** provided *E/Z* ratios of 9.4 and 9.3, respectively. The complexes **143**–**147** displayed also better diastereoselectivities in the dRRM reaction of **131** (Scheme 12) than the commercial catalysts **GII-SIMes**, **HGII-SIMes** and **IndII-SIMes**. The synthesis of indenylidene and Hoveyda-type complexes bearing *N*-phenylpyrrole and *N*-phenylindole moieties on their NHCs was also attempted [48]. Most of them revealed difficult to prepare and unstable apart from the Hoveyda-type complexes **148** and **149** (Figure 28).

These two systems were tested in standard RCM and CM reactions and complex **148** with a perbrominated *N*-phenylpyrrole moiety revealed as more stable and active than its parent catalyst **149**. Both complexes were found completely inactive in RCM at room temperature, becoming active only at higher temperature (80 °C). Computational studies suggested that the rarely occurring phenyl–ruthenium intramolecular interactions are responsible for lower stability and slower reaction initiation.

### Ruthenium catalysts coordinated with *N*-trifluoromethyl benzimidazolidene NHCs

**Figure 29:**
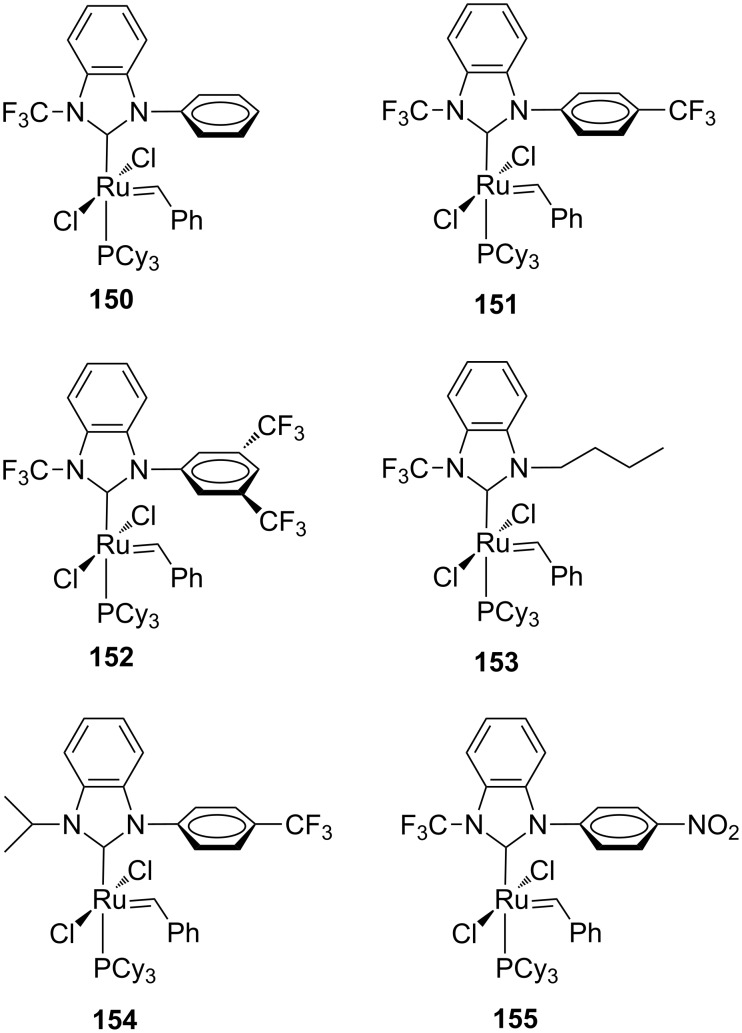
Grubbs-type complexes with *N*-trifluoromethyl benzimidazolidene NHCs **150**–**153**, **155** and *N*-isopropyl benzimidazolidene NHC **154**.

With the goal to develop chemoselective catalysts, ruthenium complexes containing unsymmetrical *N*-trifluoromethyl NHCs were introduced by Togni et al. (**150**–**152**, Figure 29) [49].

**Scheme 13:**
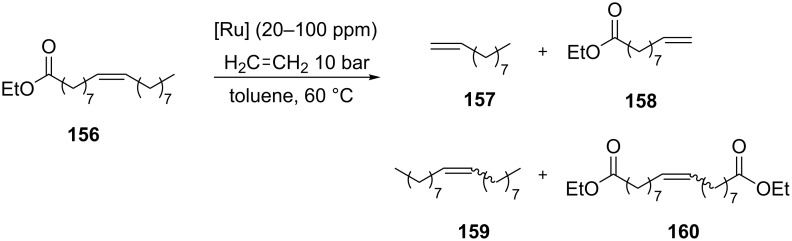
Ethenolysis of ethyl oleate **156**.

The presence of one *N*-trifluoromethyl substituent was supposed to impart positive effects on the catalytic performance, influencing both electronic and steric properties of the NHC ligand. Indeed, as already underlined, in symmetrical NHC ruthenium complexes with fluorinated *N*-aryl groups previously reported by Grubbs, a Ru–F interaction was considered as responsible for the observed enhanced metathesis activity [15]. X-ray crystallographic analysis of complexes **150**, **151** and **152** showed a Ru–F interaction in the solid state. All the catalysts were tested in benchmark RCM and CM reactions, where they displayed no improved performances compared to the commercial **GII-SIMes** catalyst. On the other hand, they showed a remarkable chemoselectivity (up to 97%) in the alternating copolymerization of norbornene (**46**) and cyclooctene (**47**). Moreover, in the ethenolysis of ethyl oleate (**156**, Scheme 13), they exhibited good selectivities (80–90%) for the formation of desired terminal olefins **157** and **158**.

Catalyst **154** containing an *N*-isopropyl group (Figure 29), which is considered to be sterically equivalent to the *N*-trifluoromethyl group, disclosed a substantially lower selectivity in both alternating copolymerization and ethenolysis reaction, underlining that the electronic effect determined by the strongly electron-withdrawing CF_3_ group and/or a Ru–F interaction are the key factors for achieving a high selectivity in these transformations and, more general, could be used for modulating catalyst properties.

**Scheme 14:**
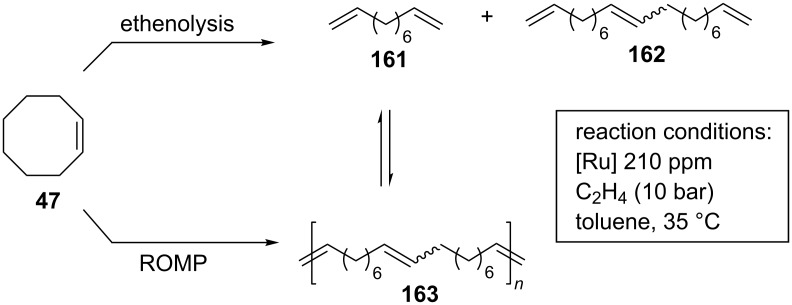
Ethenolysis of *cis*-cyclooctene (**47**).

In another contribution by Coperet, Sigman and Togni, *N*-CF_3_ complexes **150**–**155** (Figure 29) were tested for the ethenolysis of cyclic olefins to selectively form α,ω-dienes, along with other 23 Ru benzylidene complexes featuring NHC ligands that differ in steric and electronic properties [50]. It is worth to underline that this transformation mediated by ruthenium initiators is less well investigated, presumably as a consequence of the high activity of ruthenium catalysts toward the competitive ROMP that is leading to low yields of terminal dienes. Among all the investigated systems, *N*-CF_3_ complex **153** emerged as the best performing catalyst in the ethenolysis of *cis*-cyclooctene (**47**), giving 96% conversion of cyclooctene and 53% selectivity for the ethenolysis product **161** (Scheme 14). Furthermore, catalyst **153** showed no detectable formation of poly(COE) (**163**) via ROMP in the absence of ethylene. On the contrary, the benchmark catalyst **GII-SIMes** displayed only 12% selectivity for the desired product, giving predominantly poly(COE).

Due to its superior activity, complex **153** was also investigated in the ethenolysis of more challenging substrates such as norbornene derivatives, which typically are among the most popular ROMP monomers because of their high ring strain. The efficient synthesis of valuable functionalized α,ω-dienes was thus accomplished in useful yields (>70%).

In order to explain the selectivity observed in the ethenolysis of cyclic olefins, steric and electronic descriptors of the NHC ligands obtained computationally were evaluated. The main role in controlling selectivity was ascribed to the π-acceptor ability of the NHC ligand that becomes more important with dissymmetric NHCs bearing an *N*-CF_3_ group and drives the relative rate of degenerate metathesis and selectivity in ethenolysis of cyclic olefins.

### Ruthenium catalysts coordinated with backbone substituted *N*-alkyl, *N’*-aryl NHCs

Substitution at the backbone positions of the NHC framework has represented a remarkable advancement in the design of ruthenium olefin metathesis catalysts, due to the significant effects exerted on complexes' stability, reactivity and selectivity [51].

**Figure 30:**
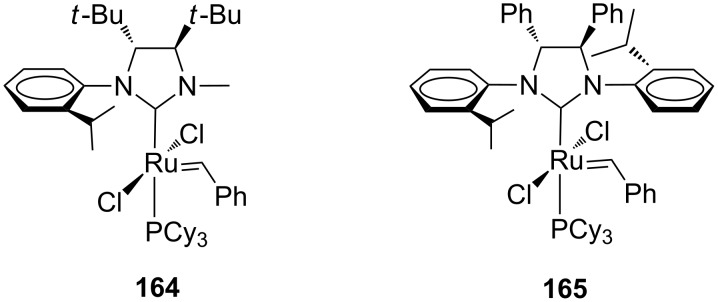
Grubbs-type *C*_1_-symmetric (**164**) and *C*_2_-symmetric (**165**) catalysts with a backbone-substituted NHC.

The first example of *C*_1_-symmetric ruthenium catalyst bearing a backbone-substituted *N*-alkyl, *N'*-aryl NHC ligand was reported by Collins et al. in 2007 (**164**, Figure 30) [52]. This complex represented an evolution of the chiral *C*_2_-symmetric system previously proposed by Grubbs (**165**, Figure 30) [53], in which the replacement of the phenyl groups on the backbone with the more encumbered and electron-donating 1,2-di-*tert*-butyl units was made with the hope to enhance reactivity and enantioselectivity in Grubbs-type olefin metathesis catalysts. Moreover, in order to reduce the whole ligand’s bulkiness which could have hampered attempts to prepare the catalyst, one of the *N*-aryl substituents was replaced with the smaller methyl group.

**Figure 31:**
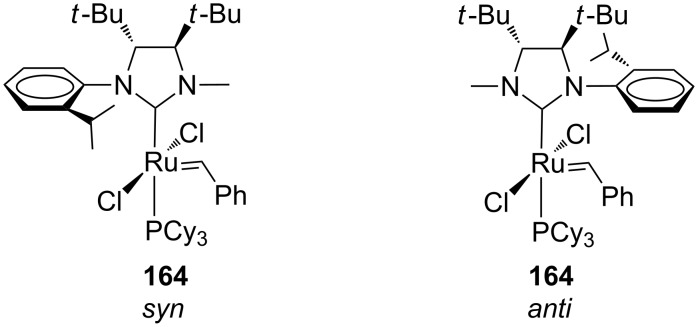
Possible *syn* and *anti* rotational isomers of catalyst **164**.

Complex **164** was obtained in poor yield (30%) and characterized through NOE and X-ray analysis, revealing the exclusive formation of the rotational isomer in which the *N*-methyl lies over the carbene unit (the *syn* isomer, Figure 31).

**Scheme 15:**
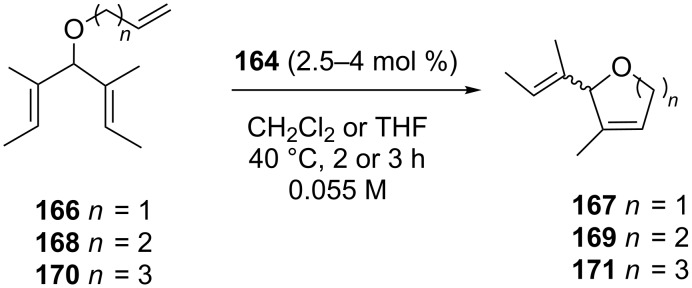
ARCM of substrates **166**, **168** and **170**.

The catalytic performances of **164** were tested in the asymmetric ring-closing metathesis (ARCM) of prochiral trienes **166**, **168** and **170** (Scheme 15, Table 6) [52,54] achieving enantiomeric excesses (ee) that were generally lower with respect to those obtained with the *C*_2_-symmetrical analogue **165** [55] (Table 6).

**Table 6:** ARCM of prochiral trienes **166**,**168**, and **170** promoted by catalysts **164** and **165**.

entry	catalyst	substrate	additive	ee (%)	conv (%)

1^a^ 2^b^ 3^b^	**164**	**166**	none NaBr NaI	82 68 48	>98 >98 >98
4^a^ 5^b^ 6^b^	**164**	**168**	none NaBr NaI	28 34 42	>98 >98 41
7^a^ 8^b^ 9^b^	**164**	**170**	none NaBr NaI	60 64 –	>98 93 –
10^a^ 11^b^	**165**^c^	**166**	none NaI	35 90	>98 >98
12^b^	**165**^c^	**168**	NaI	90	>98
13^b^	**165**^c^	**170**	NaI	85	5

^a^Catalyst 2.5 mol %, solvent CH_2_Cl_2_; ^b^catalyst 4 mol %, solvent THF [54]; ^c^[55].

The size of the ring formed was found to have a crucial influence on the enantioselectivity of the reaction with the enantiomeric excesses decreasing when passing from five to six and seven-membered rings (Table 6, entries 1, 4 and 7). The use of halide additives such as NaBr and NaI was also found to be dependent on the size of the ring formed, affecting both conversions and enantiomeric excesses with controversial results (Table 6). It should be underlined that the ambiguous halide influence constitutes a relevant difference between **164** and **165**. In fact, for the latter, the employment of halide additives had always a beneficial effect on the enantioselectivity [55].

The product ring size dependence observed in the desymmetrization of **166**, **168** and **170** with **164** was explained considering that an NHC rotation is possible during the catalytic cycle and that **166**, **168 and 170** should have different relative rates of cyclization. If the cyclization is slow, for instance in the case of seven-membered ring alkenes, an NHC rotation could occur during the catalytic cycle, thus determining a decrease of the enantiomeric excesses.

**Figure 32:**
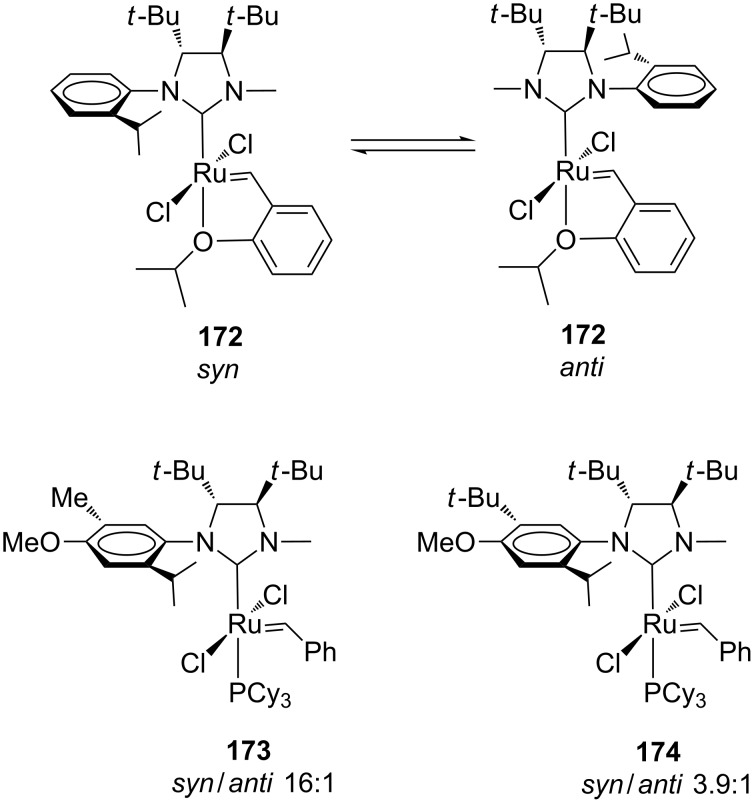
Hoveyda (**172**) and Grubbs-type (**173**,**174**) backbone-substituted *C*_1_-symmetric NHC complexes.

Rotation of the NHC ancillary ligand was detected in the case of **172**, the Hoveyda-type analogue of **164** (Figure 32), for which a room temperature interconversion between *syn* and *anti* rotamers, observed at a ratio of 7.8:1, was revealed by NOE experiments. Surprisingly, despite such rotation the reactivity profiles and the enantioselectivities observed for **164** and **172** in the desymmetrization of **166** and **170** were comparable. This suggested that the reaction occurs faster when the *N*-methyl group is *syn* to the ruthenium–carbene than when the *N*-aryl group is located *syn* to the ruthenium–carbene moiety.

In order to try suppressing the NHC rotation during the catalytic cycle, catalysts **173** and **174**, possessing additional substituents on the *N*-aryl group, were synthesized in moderate yields (42–44%, Figure 32). Both complexes were isolated as a mixture of rotamers, with a prevalence of the *syn* isomer and no interconversion between the *syn*/*anti* rotational isomers was detected at room temperature [54]. The catalytic behaviors of **173** and **174** were tested in a series of model ARCM reactions and similar or improved performances with respect to **164** and **172** were noticed, suggesting that the significant reactivity could result from the major *syn* isomer.

**Scheme 16:**
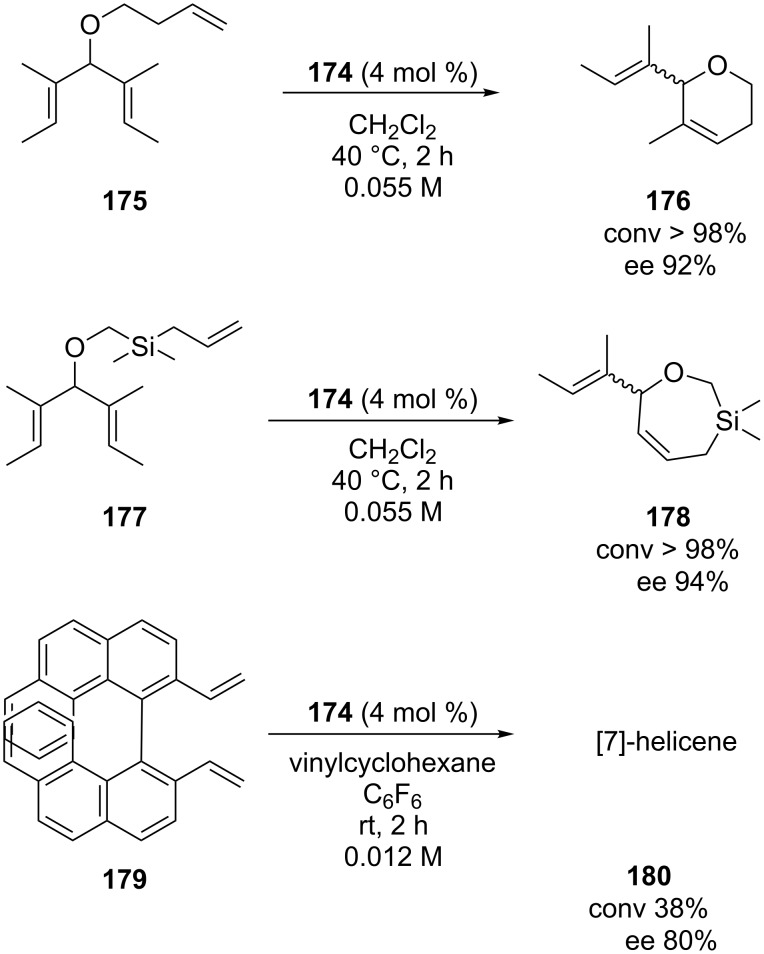
ARCM of **175**,**177** and **179** with catalyst **174**.

It is noteworthy that complex **174** was found to be very competent also in cyclizations to form six and seven-membered ring olefins (**175** and **177**, Scheme 16), conversely to the other *C*_1_-symmetric systems previously reported. On the other hand, coherently with **164** and **172**, the best results were achieved without the use of any halide additive.

**Figure 33:**
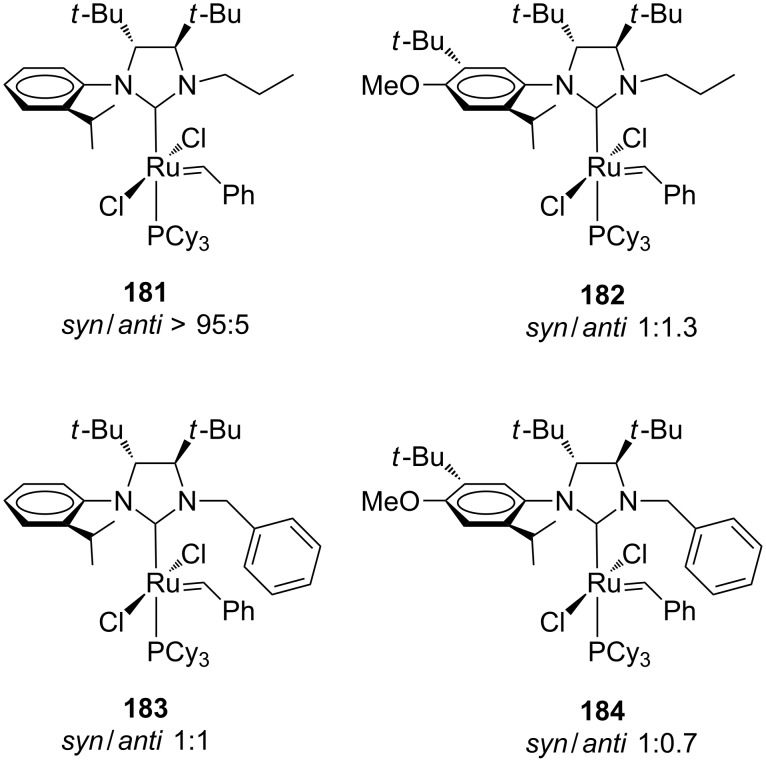
Grubbs-type *C*_1_-symmetric NHC catalysts bearing *N*-propyl (**181**, **182**) or *N*-benzyl (**183**, **184**) groups on the NHC.

The unsymmetrical NHC catalysts **164**, **173** and **174** were also examined in the asymmetric synthesis of [7]helicene (**180**). Among them, complex **174** exhibited the highest degree of selectivity, leading to the desired product with an enantiomeric excess of 80% [56]. An extension of this study, which examined the effect of the nature of the *N*-alkyl group on the complexes' efficiencies, was published a few years later by the same group [57]. In this paper, new *C*_1_-symmetric NHC ruthenium catalysts **181**–**184** bearing the more encumbered *N*-propyl or *N*-benzyl substituents were presented. All catalysts were obtained as a mixture of *syn*/*anti* rotational isomers (Figure 33).

**Scheme 17:**
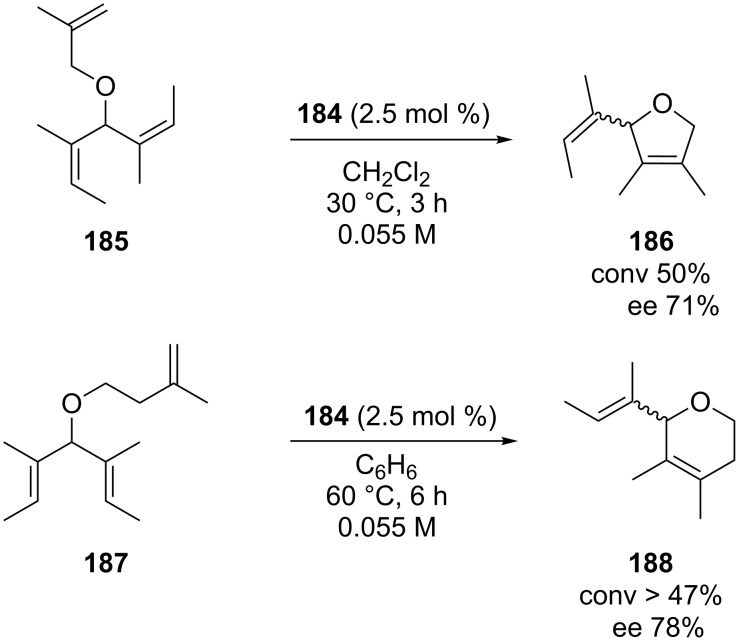
ARCM of **185** and **187** promoted by **184** to form the encumbered alkenes **186** and **188**.

The catalytic efficiency of these complexes was generally lower with respect to their *N*-methyl analogues, both in terms of reactivity and enantioselectivity. However, despite this disadvantage, they showed an improved thermal and solution stability which allowed their application also in the ARCM forming tetrasubstituted alkenes, a reaction never examined so far with this family of complexes [58]. In particular, using a sample of catalyst **184** enriched in the *anti* rotational isomer (*syn*/*anti* 1:8), the hindered cycloolefins **186** and **188** were obtained with enantiomeric excesses of 71 and 78%, respectively (Scheme 17).

**Figure 34:**
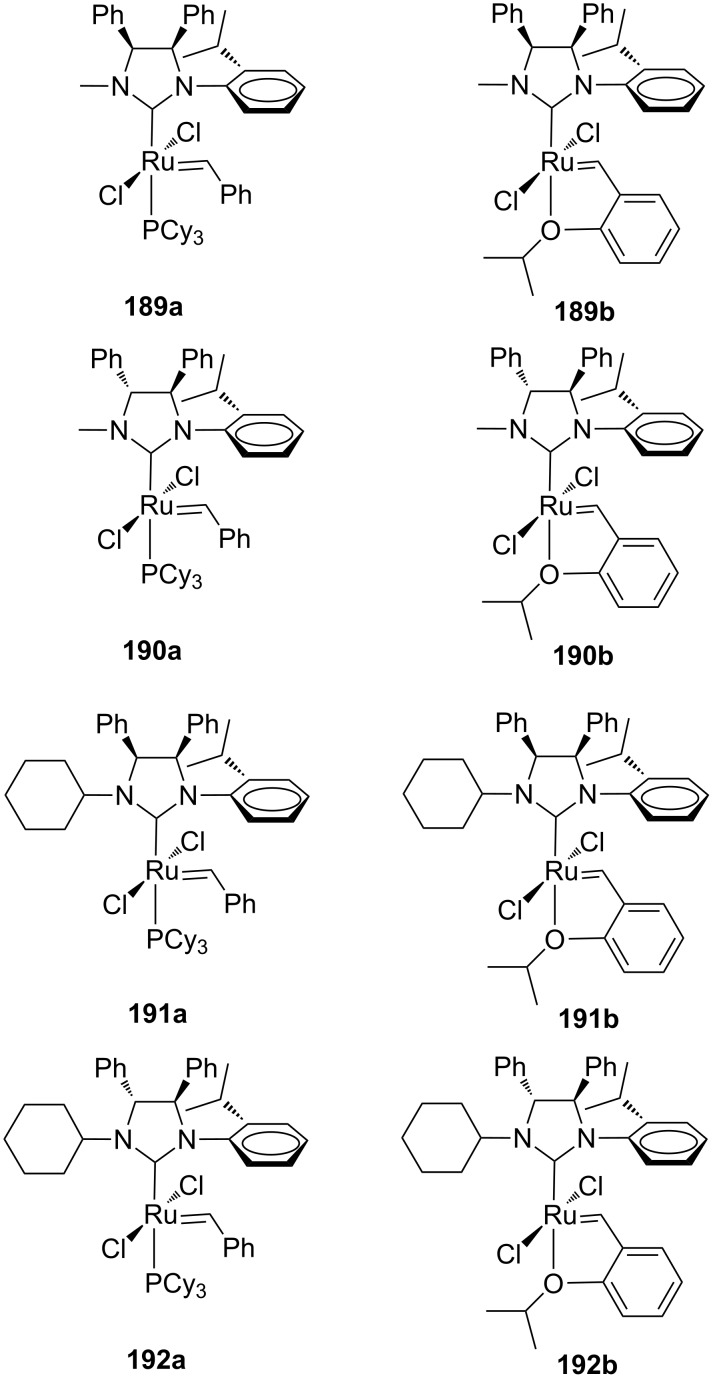
*N-*Alkyl, *N’*-isopropylphenyl NHC ruthenium complexes with *syn* (**189**, **191**) and *anti* (**190**, **192**) phenyl groups on the backbone.

More recently, Grisi and co-workers investigated new Grubbs-type *C*_1_-symmetric catalysts bearing methyl or cyclohexyl as the *N*-alkyl group and two phenyl units in *syn* or *anti* relative configuration on the backbone positions (**189**–**192**, Figure 34) [59–60]. These complexes were tested in several model RCM, ROMP and CM transformations and the size of the *N*-alkyl group and the backbone configuration seemed to determine the different catalytic behaviors. The most significant reactivity differences between catalysts having *syn* or *anti* phenyl groups on the backbone were observed in the presence of an *N*-cyclohexyl substituent. In particular, the *N*-cyclohexyl *anti* catalysts **192a** and **192b** showed high efficiencies in almost all tested metathesis transformations, especially in the most challenging RCM reactions of hindered diolefins in which they rival the commercial second generation Grubbs and Hoveyda–Grubbs catalysts. On the other hand, in the CM of **13** and **14** (Scheme 4), *syn* catalysts **191a** and **191b** gave the most interesting results, leading to the desired cross product **15** in a lower *E/Z* ratio with respect to the *anti* congeners **192a** and **192b** (*E/Z* = 3.6 and 8.5 with **191a** and **192a**, respectively; *E/Z* = 2.6 and 7.6 with **191b** and **192b**, respectively).

The effect of the NHC backbone configuration on the catalytic properties has been justified considering a more electron-donating nature of the *anti* ligand with respect to the *syn* ligand, as suggested by experimental and theoretical studies on the steric and electronic properties of *N*-cyclohexyl, *N*’-isopropylphenyl NHC ligands of **191** and **192** evaluated using the corresponding rhodium complexes [60].

**Figure 35:**
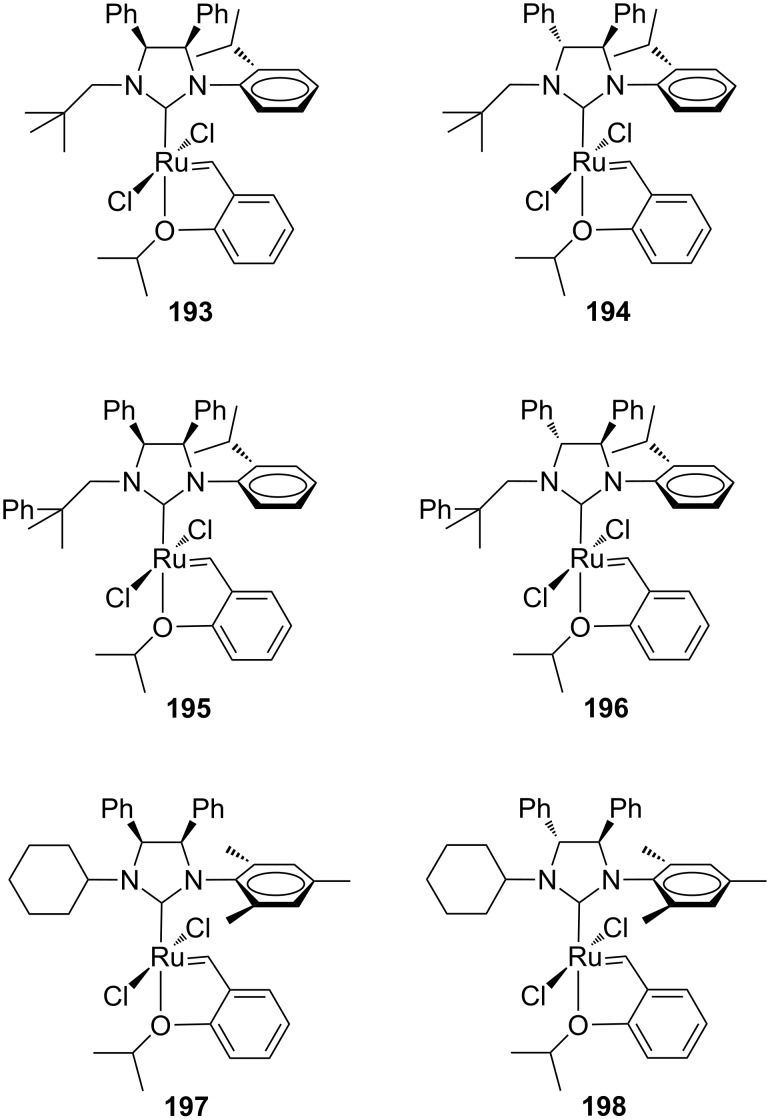
Hoveyda-type complexes **193**–**198** bearing *N-*alkyl, *N’*-aryl backbone-substituted NHC ligands.

A development of this study, which considered the utilization of other *N*-alkyl (neopentyl and neophyl) and *N*-aryl (mesityl) substituents, was published later [61]. Among these novel Hoveyda-type catalysts **193**–**198** (Figure 35), **198** was of particular interest due to its excellent thermal stability in solution and to the high efficiency in the ethenolysis of ethyl oleate (**156**, Scheme 13). In this reaction, performed under neat conditions at 50 °C and at a catalyst loading of 100 ppm, **198** gave up to 90% selectivity towards ethenolysis products **157** and **158** with a TON of 4400. At a lower catalyst loading (20 ppm), the same catalyst showed 83% selectivity with a TON of 7500, thus giving the best result reported up to now for ethenolysis reactions performed with *N*-alkyl, *N′-*aryl NHC ruthenium catalysts.

**Scheme 18:**
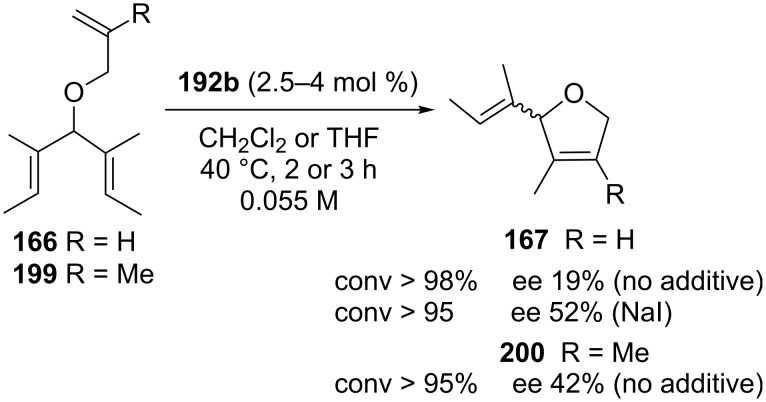
ARCM of **166** and **199** promoted by **192b**.

All the aforementioned catalysts with an *anti* NHC backbone configuration (**190**, **192**, **194**, **196** and **198**) were tested in model ARCM and AROCM reactions displaying moderate enantioselectivities [60–61]. In the ARCM of **166**, differently from the other *C*_1_-symmetric catalysts reported by Collins [52,54], enantiomeric excesses were found to increase with the use of the halide additive. Interestingly, a pronounced efficiency towards the ring closing of the hindered alkene **199** was also observed (Scheme 18).

**Figure 36:**
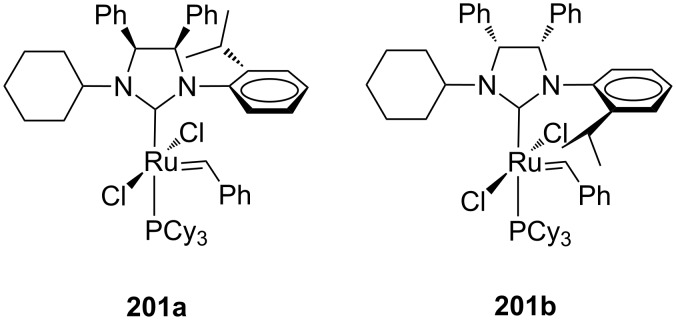
Enantiopure catalysts **201a** and **201b** with *syn* phenyl units on the NHC backbone.

In another contribution, the same group extended the feasibility in asymmetric metathesis transformations also to *C*_1_-symmetric NHC catalysts bearing *syn*-related phenyl substituents on the backbone, that were obtained for the first time in an enantiopure form (**201a** and **201b**, Figure 36) [62]. These complexes were tested in model ARCM of trienes **166** and **199** showing moderate enantioselectivities (14–44% ee).

### Ruthenium catalysts coordinated with backbone monosubstituted *N*-aryl, *N’-*aryl NHCs

**Figure 37:**
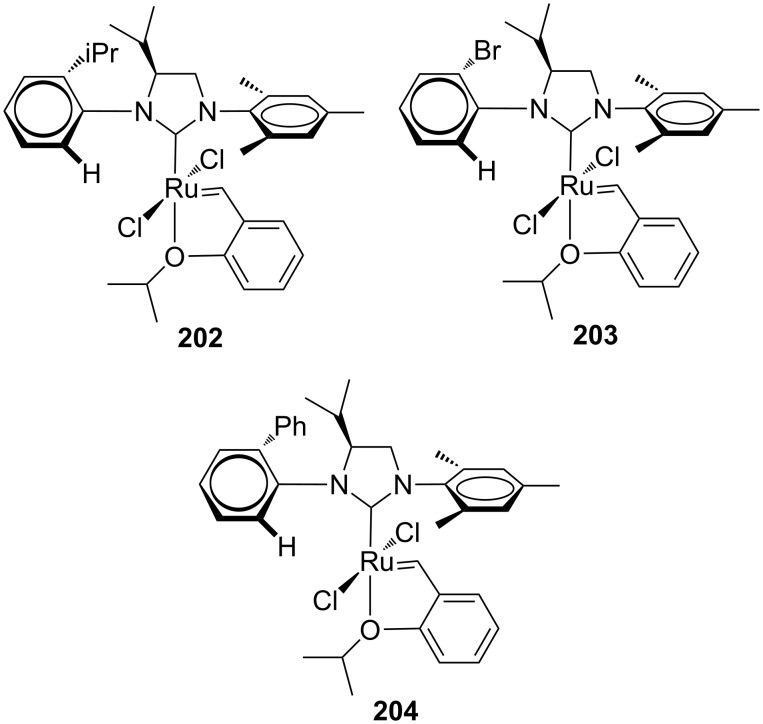
Backbone-monosubstituted catalysts **202**–**204**.

In 2010, Blechert and co-workers synthesized a new type of chiral NHC ruthenium catalysts containing a monosubstituted backbone and two different *N*-aryl groups (**202**–**204**, Figure 37) [63]. The idea behind this new category of compounds lied in the possibility of an efficient transfer of chirality from the backbone group to the metal center through a significant twisting of the monosubstituted arene unit. Additionally, the presence of the flat mesityl segment as the other *N*-aryl substituent could avoid steric hindrance reducing the reactivity.

The catalysts **202**–**204** were tested in model ARCM and AROCM reactions. In the latter transformation, they were found to be highly efficient showing both excellent enantioselectivity and *E*-selectivity. In the AROCM of **75** with styrene (Scheme 8, reaction performed at −10 °C using 5 equiv styrene and 1 mol % of the catalyst), complex **204** gave the desired product **76** in >98% conversion, 93% ee and *E*/*Z* ratio > 30:1.

**Figure 38:**
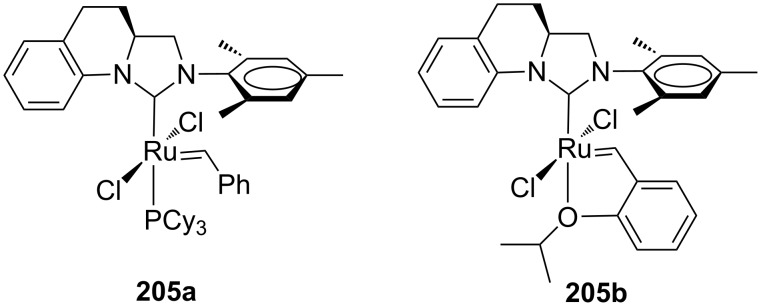
Grubbs (**205a**) and Hoveyda-type (**205b**) backbone-monosubstituted catalysts.

Pursuing on this concept, the same group subsequently published novel chiral backbone-monosubstituted NHC complexes in which a bridge connecting the *N*-aryl group and the backbone unit makes aryl rotation no longer possible, thus creating a rigid environment in the surroundings of the alkene coordination sphere (**205a**,**b**, Figure 38) [64].

**Scheme 19:**
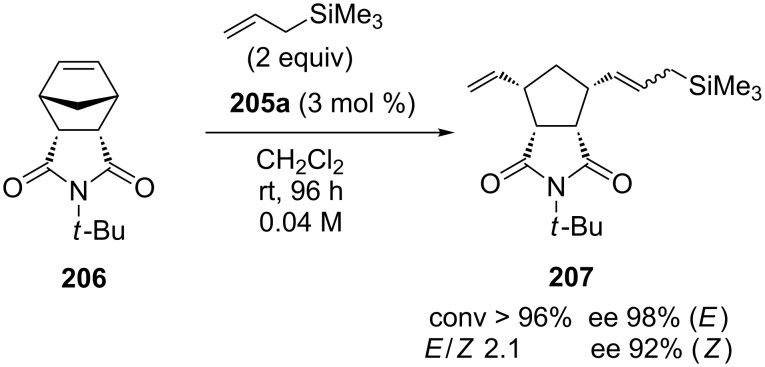
AROCM of **206** with allyltrimethylsilane promoted by catalyst **205a**.

The performances of these catalysts in AROCM transformations were comparable with those of the congeners **202**–**204** albeit they showed a lower *E*-selectivity. These systems were successfully employed for the first time in the AROCM of **206** with allyltrimethylsilane. Indeed, using catalyst **205a**, both *E* and *Z* geometric isomers of the desired cross product **207** were obtained in a high degree of enantioselectivity (Scheme 19).

## Conclusion

In the last decades, a wide array of olefin metathesis ruthenium catalysts coordinated with monodentate unsymmetrical N-heterocyclic diaminocarbene ligands have been developed. The introduction of this class of second generation catalysts, especially those containing alkyl, aryl substituted NHCs, has offered new opportunities for various metathesis applications, giving access, for instance, to highly selective alternating ring-opening metathesis polymerization, ethenolysis reactions or self metathesis of α-olefins. Both steric and electronic properties of the unsymmetrical NHCs appear to influence stability, activity and selectivity of the resulting ruthenium complexes. Therefore, the possibility to further modify the NHC ligand architectures creating new steric and electronic environments around the ruthenium center represents one of the most appealing topic on which research efforts should be focused. The development of tailor-made unsymmetrical NHC ruthenium systems is desirable to improve the efficiency in targeted metathesis reactions of not only academic but also industrial interest.
